# Supplementary material for: Dynamics of the Coreceptor-LCK Interactions during T Cell Development Shape the Self-Reactivity of Peripheral CD4 and CD8 T Cells
Source: Cell Rep. 2019 Feb 4;30(5):1504–1514.e7. doi: 10.1016/j.celrep.2020.01.008 (PMC7003063; doi:10.1016/j.celrep.2020.01.008)
Supplement: Document S1. Figures S1–S5, Table S1, and Data S1 [file mmc1.pdf]

Cell Reports, Volume *30*

Supplemental Information

Dynamics of the Coreceptor-LCK Interactions during T Cell Development Shape the Self-Reactivity of Peripheral CD4 and CD8 T Cells

Veronika Horkova, Ales Drobek, Daniel Mueller, Celine Gubser, Veronika Niederlova, Lena Wyss, Carolyn G. King, Dietmar Zehn, and Ondrej Stepanek

Figure S1

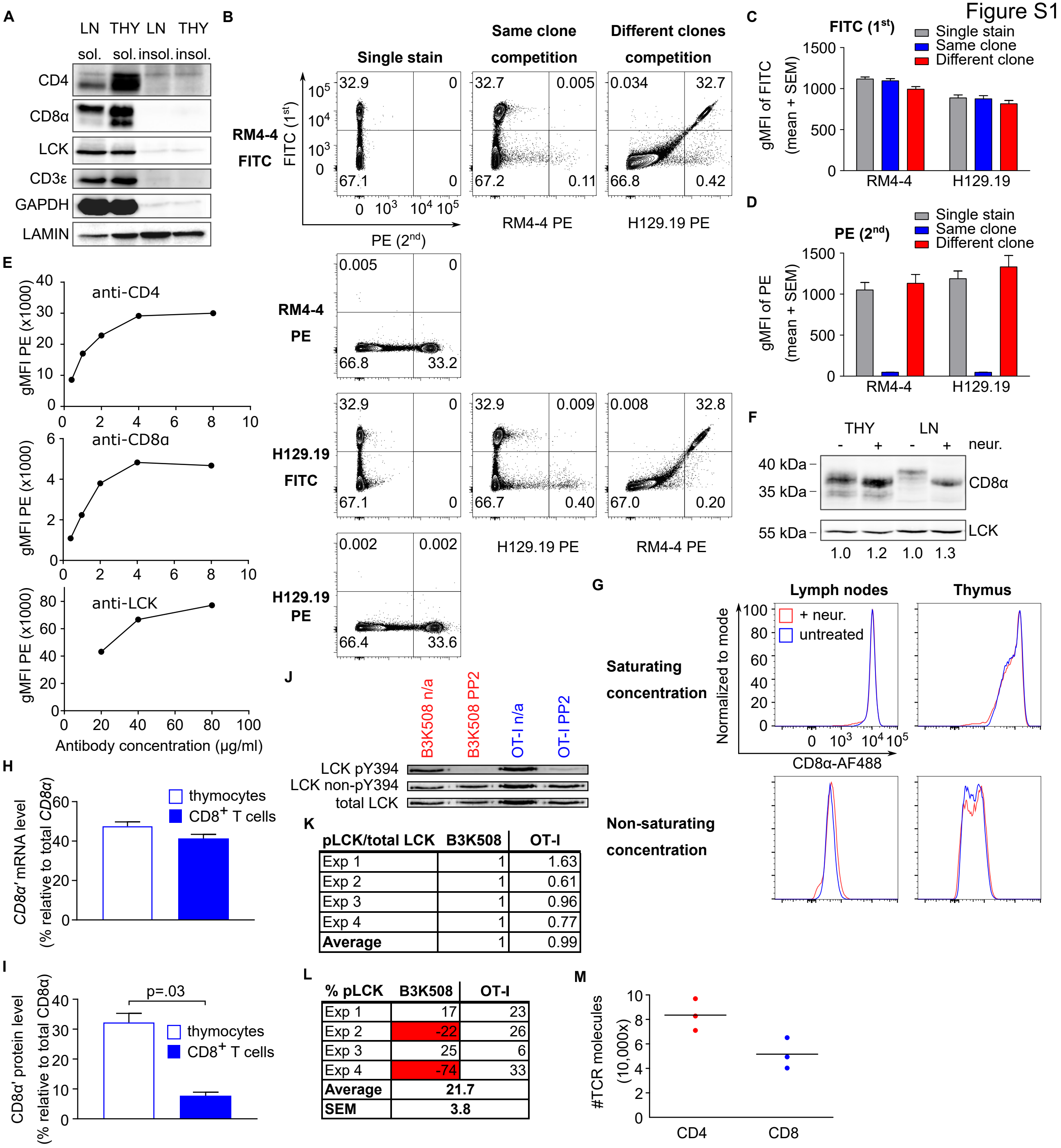

K

pLCK/total LCK	B3K508	OT-I
Exp 1	1	1.63
Exp 2	1	0.61
Exp 3	1	0.96
Exp 4	1	0.77
Average	1	0.99

L

% pLCK	B3K508	OT-I
Exp 1	17	23
Exp 2	-22	26
Exp 3	25	6
Exp 4	-74	33
Average	21.7	
SEM	3.8	

Fig. S1. Related to Figure 1.

(A) Soluble and insoluble fraction of lysate (1% NP-40S) from full LN and thymus were analyzed by immunoblotting. The membranes were stained with antibodies specific for CD4 (D7D2Z), CD8α (D4W2Z), LCK (3a5), CD3ε (goat polyclonal), GAPDH (rabbit polyclonal), LAMIN B1 (119D5-F1) (B-D) The LN T cells were stained first with FITC-conjugated antibodies (H129.19 or RM4-4). After the antibody was washed out the cells were stained with PE-conjugated antibodies specific for CD4 (H129.19 or RM4-4) in all 4 combinations. To address whether these two antibody clones compete with each other, we compared the fluorescence intensities from single stained cells, cell stained sequentially with the same antibody clone coupled to two different fluorophores, and cells stained sequentially with two different clones coupled to two different fluorophores. (B) A representative experiment out of 3 independent experiments. (C) gMFI levels of FITC. Mean + SEM, n = 3 mice (D) gMFI levels of PE. Mean + SEM, n = 3 mice. (E) Titration of antibodies used for the analysis of FC-IP experiments. A representative experiment out of 2 independent experiments is shown. (F) Thymocytes and peripheral T cells were treated with neuraminidase or not and analyzed by immunoblotting. The membranes were probed with antibodies specific for CD8α (D4W2Z) and LCK (3a5). Change in the anti-CD8α signal (normalized to LCK expression) relative to non-treated controls is indicated. Representative experiment out of 4 independent experiments. (G) Thymocytes and LN cells were treated with neuraminidase or not and analyzed by flow cytometry. The cells were stained with a saturating concentration of anti-CD8β-APC (4 μg/ml, clone 53-5.8), together with anti-CD8α-AlexaFluor488 (clone 53-6.7) at a saturating concentration of 20 μg/ml or a non-saturating concentration of 32 ng/ml. The CD8α signal on CD8β+ cells is shown. A representative experiment out of 2 in total. (H) Quantitative PCR analysis was performed on total thymocytes vs peripheral $CD8^+$ T cells. Mean + SEM, n = 3 mice (I) Quantitative analysis of surface CD8α' protein level in thymus and in peripheral $CD8^+$ T cells form experiments in Fig.1B-C. Mean + SEM, n = 5 mice in 4 independent experiments. (J-L) LCK was immunoprecipitated from lysates from non-treated (NT) or 20 μM PP2-treated B3K508 or OT-I peripheral LN T cells. Phosphorylation of LCK was analyzed by immunoblotting using simultaneous staining with antibodies specific for phosphorylated or non-phosphorylated Y394. The membrane was re-probed with antibody to total LCK. (J) Representative image out of 4 independent experiments is shown. (K) Signal of phospho-LCK to total LCK for B3K508 and OT-I T cells is shown. Data were normalized to B3K508 in each experiment. n = 4. (L) Percentage of phosphorylated LCK molecules in B3K508 and OT-I peripheral T cells was calculated for each experiment. For B3K508, two calculations led to negative values that were excluded from the final calculation, because such values make no biological and mathematical sense and were apparently caused by the error in the measurement. The average of the remaining 6 values was used for the estimation of the percentage of phosphorylated LCK molecules. (M) The peripheral $CD4^+$ and $CD8^+$ T cells were analyzed for their expression of CD3/TCR by flow cytometry.

Figure S2

A

TCR	Ligand	Selection*	Affinity (KD, µM)
OT-I	OVA	Negative	54*
OT-I	T4	Part. negative/ threshold	444*
OT-I	Q4H7	Positive	847*
B3K508	3K	Negative	29#
B3K508	P2A	Negative	175#
B3K508	P-1A	Part. negative/ threshold	263#

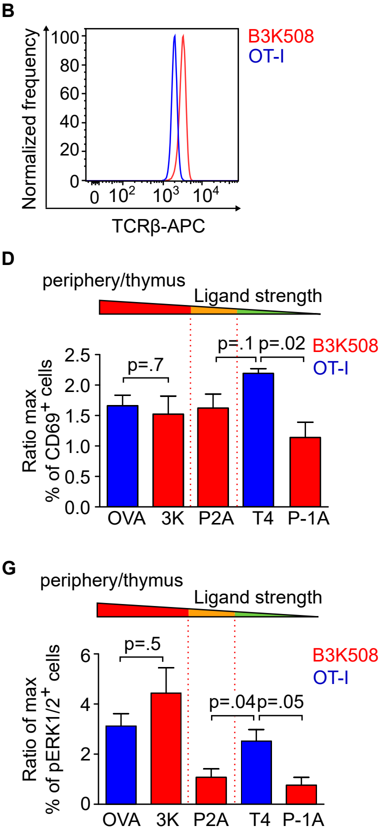

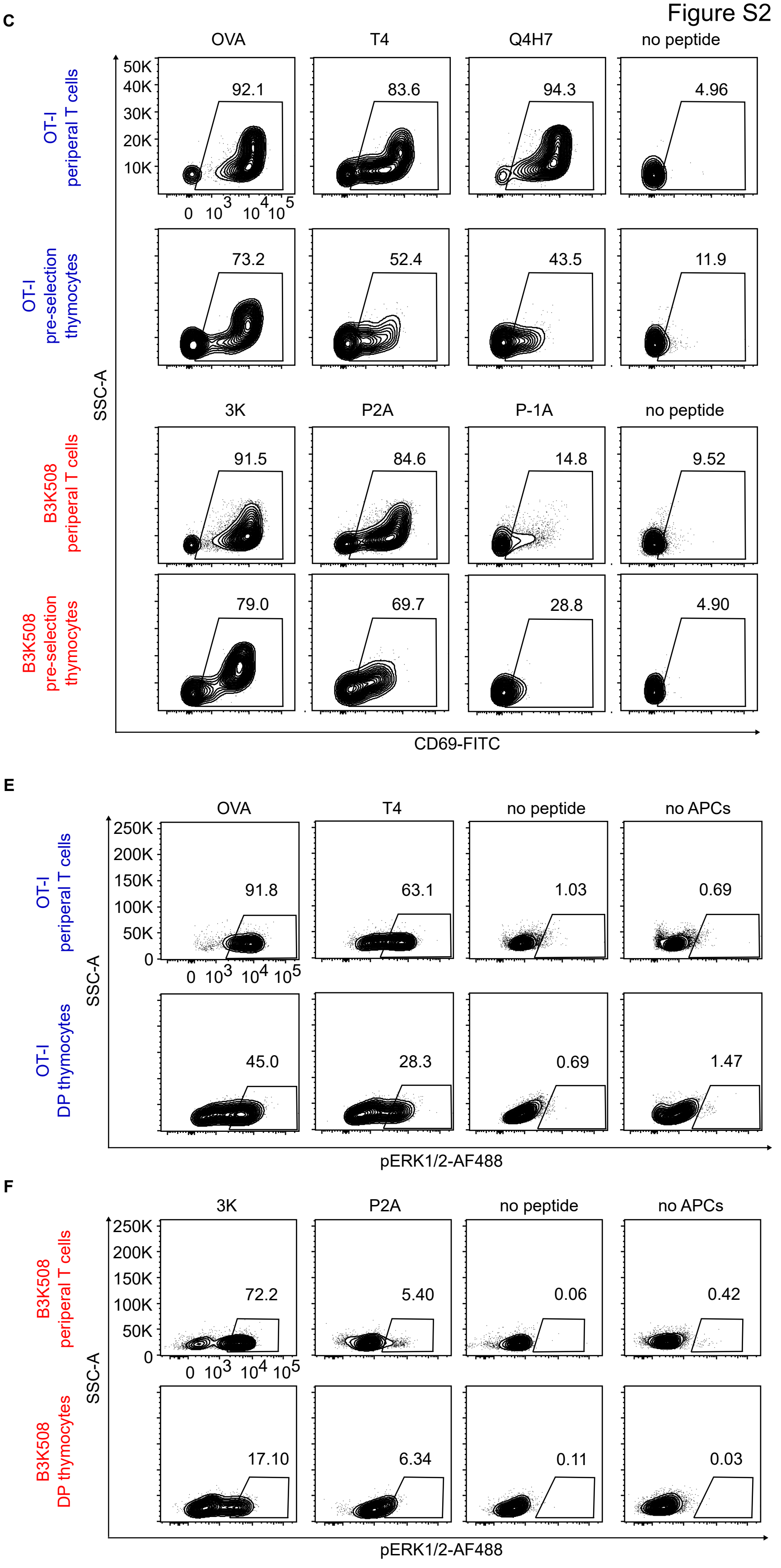

Fig. S2. Related to Figure 2.

(A) List of ligands to corresponding transgenic TCR used in Fig. 2A-D. The measured dissociation constant *(Stepanek et al., 2014) #(Huseby et al., 2006) and their ability to induce the thymic selection is denoted. (B) TCRβ expression on $CD8^+$ $CD44^-$ OT-I and $CD4^+$ $CD44^-$ B3K508 T cells. A representative experiment out of 4 in total. (C) Representative data from experiments shown in Fig. 2A are shown. (D) The ratio of maximum activation (% of $CD69^+$ cells) of the T cells in periphery vs. thymus. Mean + SEM, n = 4 mice in 4 independent experiments. (E-F) Alternative analysis of experiments shown in Figure 2. Representative data from experiments shown in Fig. 2C are shown. (G) The ratio of maximum activation (% of $CD69^+$ cells) of the T cells in periphery vs thymus. Mean + SEM. n = 6-10 mice in 6-10 independent experiments.

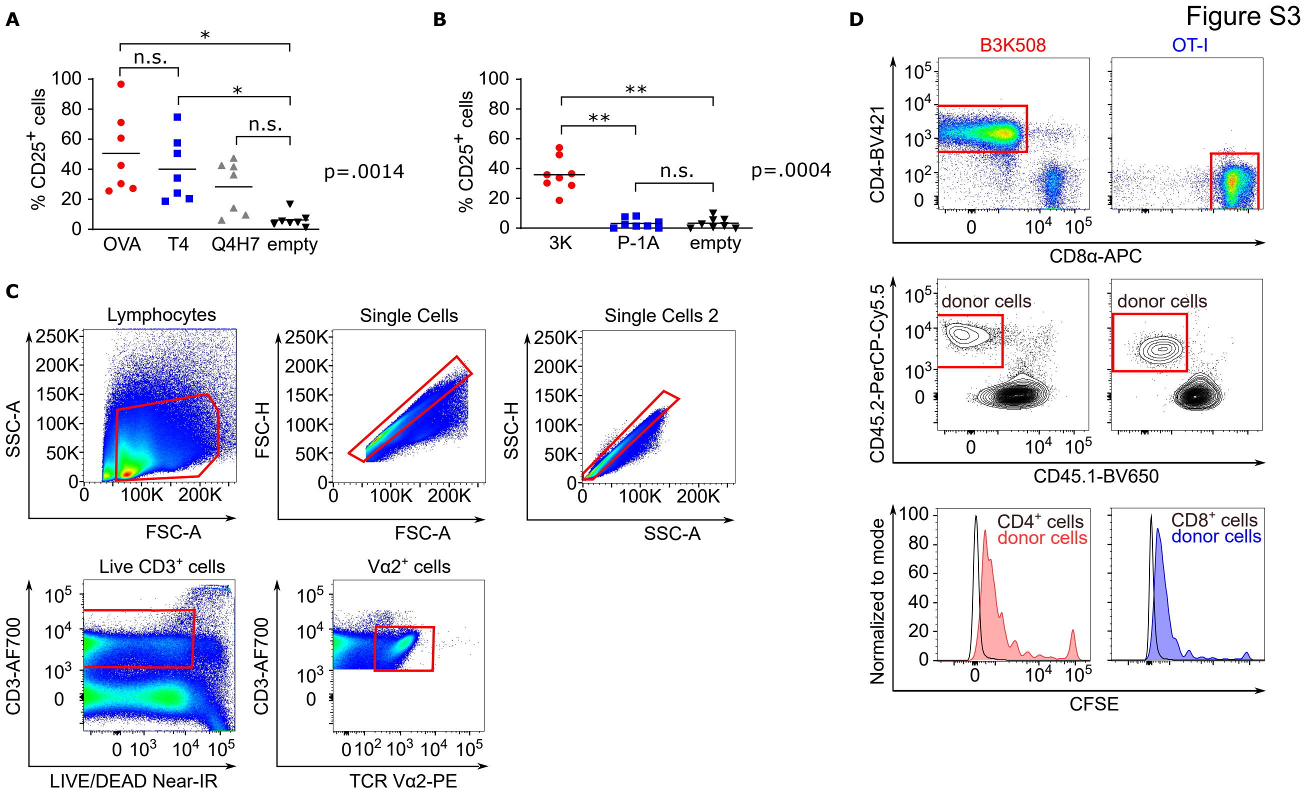

Figure S3
A
% CD25+ cells
OVA
T4
Q4H7
empty
n.s.
*
p=.0014
B
3K
P-1A
empty
**
n.s.
p=.0004
C
Lymphocytes
Single Cells
Single Cells 2
SSC-A
FSC-A
FSC-H
Live CD3+ cells
Vα2+ cells
CD3-AF700
LIVE/DEAD Near-IR
TCR Vα2-PE
D
B3K508
OT-I
CD4-BV421
CD8α-APC
donor cells
CD45.2-PerCP-Cy5.5
CD45.1-BV650
CD4+ cells
CD8+ cells
Normalized to mode
CFSE

Fig. S3. Related to Figure 3.

(A-B) Quantification of % $CD25^+$ monoclonal T cells from experiment in Fig.3A-B. Statistical analysis was performed using Kruskal-Wallis test with Dunn's Multiple Comparison post-tests (* $p<0.05$, ** $p<0.01$). (C-D) Gating strategy for the experiments in Fig.3A-B. (C) Initial gating strategy used for all samples. (D) Additional gating strategy for B3K508 (Lm-3K) or OT-I (Lm-T4) cells. The CFSE signals on donor cells are shown as histograms.

Figure S4

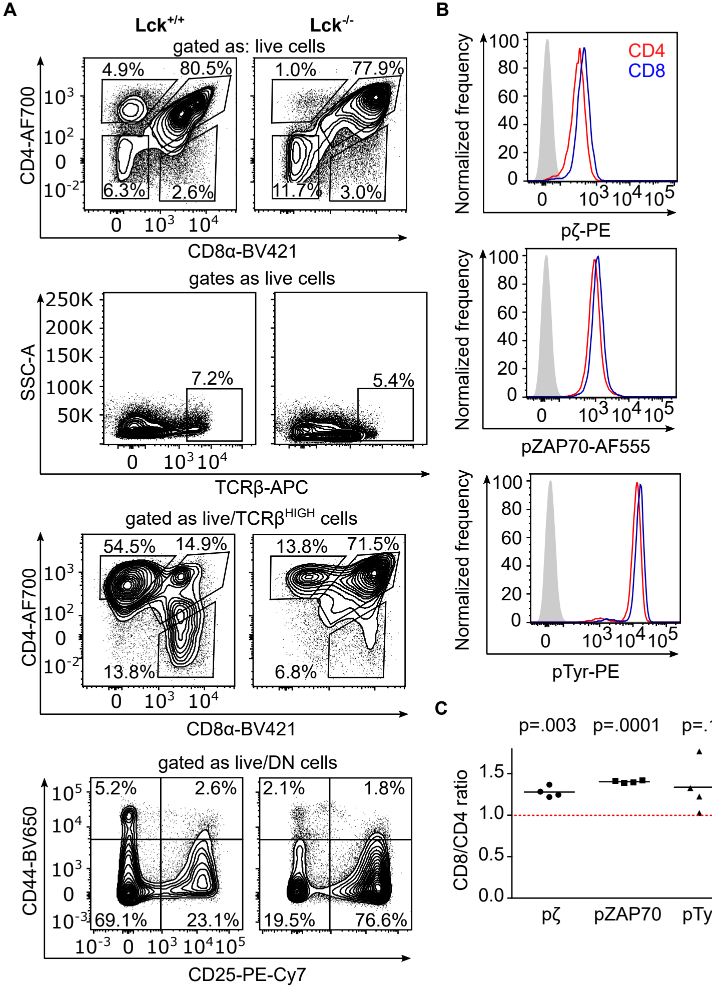
A
Lck+/+
Lck-/-
gated as: live cells
4.9%
80.5%
6.3%
2.6%
1.0%
77.9%
11.7%
3.0%
CD4-AF700
CD8α-BV421
gates as live cells
7.2%
5.4%
SSC-A
250K
200K
150K
100K
50K
TCRβ-APC
gated as live/TCRβHIGH cells
54.5%
14.9%
13.8%
13.8%
71.5%
6.8%
gated as live/DN cells
5.2%
2.6%
69.1%
23.1%
2.1%
1.8%
19.5%
76.6%
CD44-BV650
CD25-PE-Cy7
B
CD4
CD8
Normalized frequency
pζ-PE
pZAP70-AF555
pTyr-PE
C
p=.003
p=.0001
p=.1
CD8/CD4 ratio
pζ
pZAP70
pTyr

Fig. S4. Related to Figure 4.

(A) Flow cytometry analysis of thymocytes from *Lck*$^{+/+}$ and *Lck*$^{-/-}$ mice using indicated antibodies and LIVE/DEAD near-IR staining. A representative experiment out of 3 in total. (B-C) Fixed and permeabilized LN T cells from *Foxp3*-GFP mice were stained with antibodies to CD4, CD8, TCRβ, pTCRζ, pZAP70, and overall tyrosine phosphorylation and analyzed by flow cytometry. Only GFP-negative cells are shown. A representative experiment out of 3 mice in 2 independent experiments in total.

Figure S5

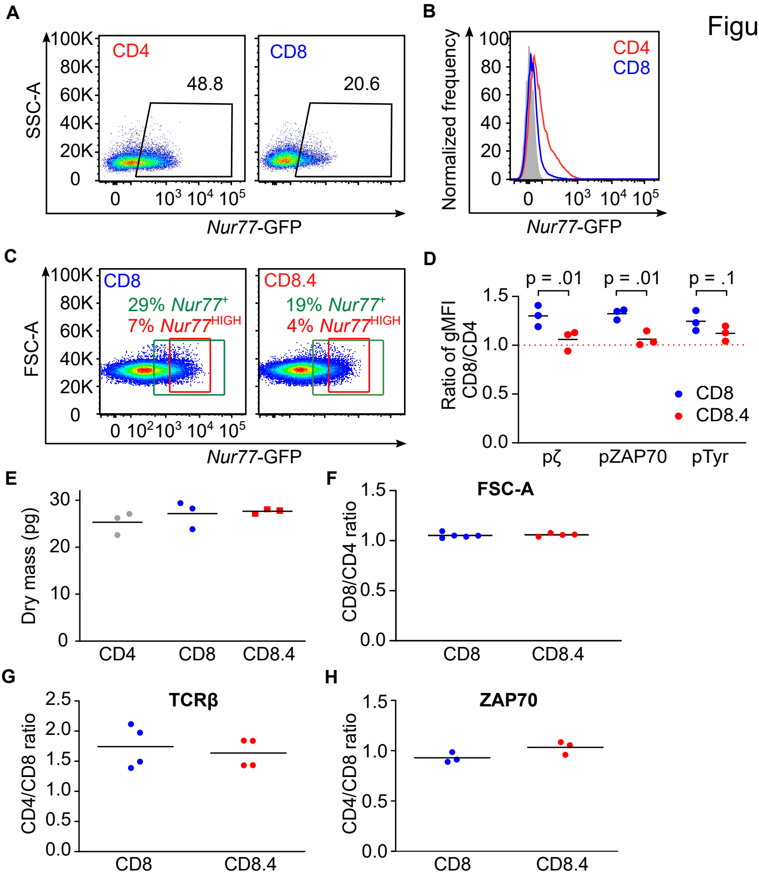

A
CD4
48.8
CD8
20.6
SSC-A
100K
80K
60K
40K
20K
0
0 10³ 10⁴ 10⁵
Nur77-GFP
B
Normalized frequency
100
80
60
40
20
0
0 10³ 10⁴ 10⁵
Nur77-GFP
C
CD8
29% Nur77⁺
7% Nur77^HIGH
CD8.4
19% Nur77⁺
4% Nur77^HIGH
FSC-A
0 10² 10³ 10⁴ 10⁵
Nur77-GFP
D
p = .01
p = .01
p = .1
Ratio of gMFI CD8/CD4
1.5
1.0
0.5
0.0
pζ
pZAP70
pTyr
CD8
CD8.4
E
Dry mass (pg)
30
20
10
0
CD4
CD8
CD8.4
F
FSC-A
CD8/CD4 ratio
1.5
1.0
0.5
0.0
CD8
CD8.4
G
TCRβ
CD4/CD8 ratio
2.5
2.0
1.5
1.0
0.5
0.0
CD8
CD8.4
H
ZAP70
CD4/CD8 ratio
1.5
1.0
0.5
0.0
CD8
CD8.4

Fig. S5. Related to Figure 5.

(A) Representative data from the CD4$^+$ and CD8$^+$ T cells gated for *Nur77*-GFP$^+$. Data out of 5 independent experiments are shown. (B) Representative data on *Nur77* expression of the CD4$^+$ and CD8$^+$ T cells. The data are the same as in Fig. S5A. (C) Representative data from CD8 WT and CD8.4 naïve T cells gated for *Nur77*-GFP$^+$ and *Nur77*-GFP^{HIGH}. (D) Independent repetition of experiment in Fig.5B performed in Prague. Statistical significance was calculated using paired T test. n = 3 mice in 3 independent experiments. (E) Peripheral LN T cells from C57Bl/6J and CD8.4 mice were analyzed on Tescan Q-Phase microscope. Dry mass of individual populations is displayed. Mean. n = 3 mice in 3 independent experiments. (F) Peripheral LN T cells from C57Bl/6J and CD8.4 mice were analyzed by flow cytometry and gated as CD44$^-$ CD4$^+$ or CD44$^-$ CD8$^+$ cells. The relative size (gMFI of FSC-A) of CD8$^+$ or CD8.4$^+$ cells to CD4$^+$ cells is displayed. Mean. n = 4-5 mice in 4-5 independent experiments. (G) Relative expression (gMFI) of *Zap70* in CD8$^+$ or CD8.4$^+$ T cells compared to CD4$^+$ T cells is shown. Mean. n = 3 mice in 3 independent experiments.

Table S1. Related to Fig. 1: Parameters for the mathematical model related to Fig.1C.

Parameter	**diffusion (D)**	**coreceptor-MHC offrate (Ku)**	**lattice spacing (l)**	**coreceptor-MHC onrate (Kb)**	**pLck coupling (f)**	**number of TCRs/3 (T)**	**Area**	**k_{on}**	**k_p**	**phospho - rylation**
Units	$\mu m^2 s^{-1}$	s^{-1}	µm	s^{-1}	%		μm^2	$\mu m^2 s^{-1}$	s^{-1}	
**CD4 DP thymocytes**	0.08	200	0.01	1000	0.051	697	26.18	0.1	5	5
**CD4 LN T cells**	0.08	200	0.01	1000	0.108	27861	26.18	0.1	5	5
**CD8 DP thymocytes**	0.08	20	0.01	1000	0.0054	697	26.18	0.1	5	5
**CD8 LN T cells**	0.08	20	0.01	1000	0.072	17203	26.18	0.1	5	5

Data S1 Related to Fig.1: Source code for ‘Lck come&stay/signal duration’ model (MatLab).

```
% Main script

% calculates Come%Stay model from given parametes using essentially 4
% conditions (can vary in coupling), the result are 4 matrices where X is
% DOSE and Y is dwell time)

%dwelltimes=zeros(100,100);
%for i=1:10 % makes an array of dwell times, always in columns from 0.05 to 20 in 0.025 steps, 1000 columns (all are the same)
    %dwelltimes(:,i)=i*0.1;
%end

%antigens=zeros(100,100); %makes an array of antigen numbers from 1 to 1000, 800 rows (all are the same)
%for j=1:100
    %antigens(j,:)=j;
%end

antigens=1:250;

sourcedata1=fopen('comenstaydata_5_6_P.txt','r'); %opens file with source data (dataDIAOTI.txt) and calls it sourcedata1
Dat=fscanf(sourcedata1,'%f', [10,4]) % reads data from the file
Cordata=Dat.';
Data1=Cordata(1,:) % condition 1
Data2=Cordata(2,:); % condition 2
Data3=Cordata(3,:); % condition 3
Data4=Cordata(4,:); % condition 4

dw1=0.86; % antigen dwell time (CD4s) 0.2 for threshold Ag, 0.86 FOR 3K
dw2=10.5; % antigen dwell time (CD8s) 0.9 for threshold Ag, 10.5 for OVA (approx. for thymus)

res1=dataprocess(Data1, dw1, antigens); % calls data process function for datasets 1-4
res2=dataprocess(Data2, dw1, antigens);
res3=dataprocess(Data3, dw2, antigens);
res4=dataprocess(Data4, dw2, antigens);

semilogy(antigens,res1, 'red:', 'LineWidth',2.5)
set(gca,'FontSize',17);

hold on % to draw all lines to the same graph
semilogy(antigens,res2, 'red', 'LineWidth',2.5)
semilogy(antigens,res3, 'blue:', 'LineWidth',2.5)
semilogy(antigens,res4, 'blue', 'LineWidth',2.5)
xlabel('Number of antigens','fontsize', 15, 'FontWeight','bold')
ylabel('# TCRs triggered','fontsize', 15, 'FontWeight','bold')
hold off
```

```
% Functions
function [lam] = markovchain ( D, Ku, l, Kb, f)
% Gives lambda parameter from an analytical Markov Chain solution
% lambda is the rate of Lck recruitment to the TCR via coreceptors
% Describes output of the coreceptor scanning mechanism
% D is a diffusion coefficient for membrane proteins, std 0.08 um^2
% x s-1
% cor_Koff is the correceptor Koff from the MHC molecule, std 20s-1 for
% CD8, 200s-1 for CD4
% l is a lattice spacing of the model, std 0.01 um
% cor_Kon is on-rate of the coreceptor-MHC interaction, std 1000s-1
lam = (f.*D.*Ku)./(D+l.^2.*Kb);
end

function [hotTCR] = dataprocess( dataValues, dwelltimeValue, antigenNumber )
% uses input data in a horizontal vector (reading from a file) and
% calculates the output of the comenstay, i.e. calculates lambda and then
% number of triggered and occupied TCRs

lambda=markovchain(dataValues(1,1), dataValues(1,2),dataValues(1,3), dataValues(1,4),dataValues(1,5))
hotTCR=comenstay(lambda, antigenNumber, dataValues(1,6), dataValues(1,7), dwelltimeValue, dataValues(1,8), dataValues(1,9), dataValues(1,10));

end

function [ R ] = comenstay( la, L, T, A, t, kon, kp, n )

%Gives abverage number of occupied and triggered TCRs at the equillibrium
%  from Stepanek et al. Cell 2014
% la is lambda (rate of Lck recruitment, calculated by markov chain model)
% L is number of antigen (pMHC) molecules) in the contact area, varied
% T is number of TCRs in the contact area
% A is the area of the T cell/APC inteface, std 26 um^2
% t is dwell-time, i.e. half-life (varied)
% kon is the on-rate of the antigen, std 0.1 um^2s-1
% kp is the phosphorylation rate, std 5s-1
% n is the required number of phosphorylation steps

%la=ones(100)*la1; %to have it in matrices (optional), change THE INPUT
%variables accordigly
%T=ones(100)*T1;
%A=ones(100)*A1;
%kon=ones(100)*kon1;
%kp=ones(100)*kp1;
%n=ones(100)*n1;
```

```
af=log(2)./(kon.*t); % Affinity, to simplify the final equation ln2/(kon x t)
koff=log(2)./t; % koff calculated

ProbTrig=la./(la+koff).*(kp./(kp+koff)).^n; %probability that a single TCR/MHC interaction leads to the TCR triggering
OccTCRs = (L/A+T/A+af-((L./A+T./A+af).^2-4*L.*T./A^2).^0.5)/2.*A; %average number of antigen occupied by MHC antigens in equillibrium, . removed when not necessary

R= ProbTrig.*OccTCRs; % number of triggered and occupied TCRs at the equilibrium, OUTPUT of the Come&Stay model
```
