# Supplementary material for: Dynamics of the Coreceptor-LCK Interactions during T Cell Development Shape the Self-Reactivity of Peripheral CD4 and CD8 T Cells
Source: Cell Rep. 2019 Feb 4;30(5):1504–1514.e7. doi: 10.1016/j.celrep.2020.01.008 (PMC7003063; doi:10.1016/j.celrep.2020.01.008)
Supplement: Document S2. Article plus Supplemental Information [file mmc2.pdf]

Cell Reports

Article

Dynamics of the Coreceptor-LCK Interactions during T Cell Development Shape the Self-Reactivity of Peripheral CD4 and CD8 T Cells

Graphical Abstract

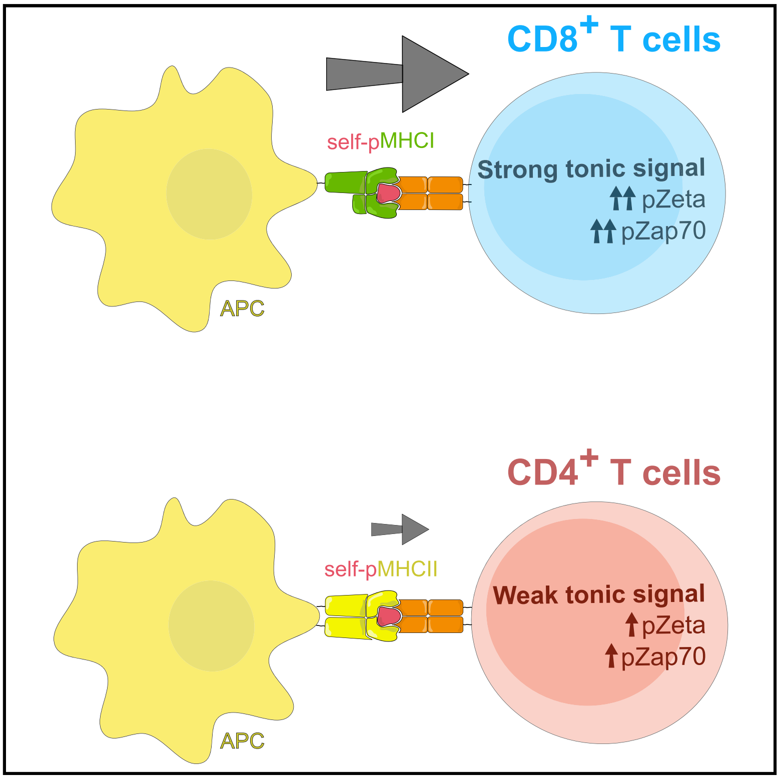

Highlights

- Coupling of CD8-LCK but not CD4-LCK increases upon T cell maturation
- Dynamics of coreceptor-LCK coupling stoichiometry establish T cell self-reactivity
- $CD8^+$ T cells are more self-reactive than $CD4^+$ T cells

Authors

Veronika Horkova, Ales Drobek, Daniel Mueller, ..., Carolyn G. King, Dietmar Zehn, Ondrej Stepanek

Correspondence

ondrej.stepanek@img.cas.cz

In Brief

Horkova et al. reveal dynamic regulation of the coreceptor-LCK interaction during T cell development, establishing the self-reactivity of mature T cells. Differences between CD8 and CD4 coreceptors cause peripheral $CD8^+$ T cells to be more self-reactive than $CD4^+$ T cells.

Horkova et al., 2020, Cell Reports *30*, 1504–1514
February 4, 2020 © 2020 The Author(s).
https://doi.org/10.1016/j.celrep.2020.01.008

CellPress

OPEN ACCESS
CellPress

Cell Reports
Article

Dynamics of the Coreceptor-LCK Interactions during T Cell Development Shape the Self-Reactivity of Peripheral CD4 and CD8 T Cells

Veronika Horkova,[1] Ales Drobek,[1] Daniel Mueller,[2] Celine Gubser,[2,3] Veronika Niederlova,[1] Lena Wyss,[2,4] Carolyn G. King,[2] Dietmar Zehn,[5] and Ondrej Stepanek[1,6,*]
[1]Laboratory of Adaptive Immunity, Institute of Molecular Genetics of the Czech Academy of Sciences, 14220 Prague, Czech Republic
[2]Department of Biomedicine, University Hospital and University of Basel, 4031 Basel, Switzerland
[3]Peter Doherty Institute, University of Melbourne, Melbourne, Australia
[4]Institute for Immunology, Biomedical Center (BMC) Munich, Ludwig-Maximilians-University, Munich, Germany
[5]Division of Animal Physiology and Immunology, School of Life Sciences Weihenstephan, Technical University of Munich, Freising, Germany
[6]Lead Contact
*Correspondence: ondrej.stepanek@img.cas.cz
https://doi.org/10.1016/j.celrep.2020.01.008

SUMMARY

Overtly self-reactive T cells are removed during thymic selection. However, it has been recently established that T cell self-reactivity promotes protective immune responses. Apparently, the level of self-reactivity of mature T cells must be tightly balanced. Our mathematical model and experimental data show that the dynamic regulation of CD4- and CD8-LCK coupling establish the self-reactivity of the peripheral T cell pool. The stoichiometry of the interaction between CD8 and LCK, but not between CD4 and LCK, substantially increases upon T cell maturation. As a result, peripheral CD8$^+$ T cells are more self-reactive than CD4$^+$ T cells. The different levels of self-reactivity of mature CD8$^+$ and CD4$^+$ T cells likely reflect the unique roles of these subsets in immunity. These results indicate that the evolutionary selection pressure tuned the CD4-LCK and CD8-LCK stoichiometries, as they represent the unique parts of the proximal T cell receptor (TCR) signaling pathway, which differ between CD4$^+$ and CD8$^+$ T cells.

INTRODUCTION

T cells are involved in most adaptive immune responses. The hallmark of T cell responses is the variability of T cell receptors (TCRs) among individual T cell clones. The interaction between the TCR and its cognate antigen (i.e., a peptide bound to major histocompatibility complex class I [MHCI] or MHCII molecules) on the surface of an antigen-presenting cell (APC) leads to the activation of the T cell and the initiation of the immune response.

There are two basic types of T cells, MHCI-restricted CD8$^+$ T cells and MHCII-restricted CD4$^+$ T cells. CD8$^+$ T cells are involved in direct killing of infected cells, whereas CD4$^+$ T cells orchestrate immune responses by acting on other immune cells. Invariant coreceptors CD4 and CD8 bind to MHCI and MHCII, respectively, to promote the TCR signaling. One of the major roles of the coreceptors is to recruit a kinase LCK to the TCR signaling complex, which, in turn, leads to the phosphorylation of the TCR-associated chains and the initiation of the downstream signaling (Artyomov et al., 2010; van Oers et al., 1996; Rudd et al., 2010; Veillette et al., 1988; Barber et al., 1989). The interaction between the coreceptor and LCK regulates the sensitivity of T cells to the antigen (Erman et al., 2006; Stepanek et al., 2014; Drobek et al., 2018).

Besides their key role in protective immunity, T cells can induce harmful autoimmunity, depending on whether they respond to foreign or self-antigens. A central mechanism establishing self-tolerance is the negative selection of highly self-reactive T cells during their maturation in the thymus. However, a certain level of self-reactivity of mature T cells is required because only the self-pMHC-restricted pool of T cells can efficiently recognize foreign antigens. This is achieved by positive selection of developing T cells with moderate reactivity to self-antigens in the thymus. The stoichiometry of the coreceptor-LCK interaction sets up the “selection window” by establishing the thresholds for positive and negative selection of developing T cells (Erman et al., 2006; Stepanek et al., 2014).

There is an increasing amount of evidence showing that the actual level of self-reactivity largely determines T cell responses to foreign cognate antigens. It has been shown that the level of self-reactivity correlates with the ability of T cells to recognize foreign antigens with high affinity (Mandl et al., 2013). A comparison of two CD4$^+$ T cell clones with identical affinity for the cognate antigen revealed that the less self-reactive clone expanded more in the primary response, whereas the more self-reactive clone dominated the recall response (Weber et al., 2012; Persaud et al., 2014). Other studies showed that priming of T cells by self-antigens enhances their subsequent responses to foreign antigens and that highly self-reactive T cells have an advantage over weekly self-reactive T cells in this respect (Fulton et al., 2015; Swee et al., 2016; Stefanová et al., 2002). Overall, the self-reactivity of T cells is beneficial for immune protection, but at the same time, it represents the risk for the onset of autoimmunity. Apparently, there is an optimal level of self-reactivity that balances these two counteracting phenomena. This optimal level of self-reactivity can be established by correct setting of the thresholds for positive and negative selection in the thymus

 © 2020 The Author(s).
This is an open access article under the CC BY license (http://creativecommons.org/licenses/by/4.0/).

and/or by eventual changes in the sensitivity of the TCR signaling machinery during T cell maturation. Considering fundamental differences in the roles of CD8$^+$ and CD4$^+$ T cells, it is very plausible that the optimal levels of self-reactivity might substantially differ between these two populations.

We and others showed that CD4-LCK and CD8-LCK binding stoichiometry is a limiting factor for signaling induced by suboptimal self-antigens in immature thymocytes (Erman et al., 2006; Stepanek et al., 2014). Moreover, we have recently revealed that the CD8-LCK binding frequency regulates tonic TCR signaling in peripheral T cells and the generation of virtual memory T cells from relatively highly self-reactive CD8$^+$ T cells (Drobek et al., 2018). However, the stoichiometry of CD4-LCK and CD8-LCK interactions in mature T cells has not been addressed in detail.

In this study, we observed that the stoichiometry of the CD8-LCK, but not CD4-LCK, interaction is dynamically regulated during development. The percentage of CD8 molecules carrying LCK is substantially higher in mature T cells than in thymocytes at the double positive (DP) stage, where the positive selection and most of the negative selection takes place. Consequently, CD8$^+$ T cells increase their responsiveness to antigens with suboptimal affinity upon maturation. Moreover, CD8$^+$ T cells are, on average, more self-reactive than CD4$^+$ T cells. Our observation seems to be a result of an evolutionary adaptation that took advantage of the different use of coreceptors by MHCI- and MHCII-restricted T cells to tune the optimal level of self-reactivity for these two subsets independently.

RESULTS

CD8-LCK Coupling Frequency Is Dynamically Regulated during T Cell Maturation

The stoichiometry of the CD4-LCK and CD8-LCK interactions has been previously analyzed using a semiquantitative method of immunoprecipitation followed by flow cytometry (FC-IP) in preselection DP thymocytes (Stepanek et al., 2014). In this study, we applied this method to mature peripheral T cells. This method is based on of the immunoprecipitation of CD4 or CD8 by using antibody-coated beads, followed by the detection of coreceptor and LCK molecules by flow cytometry. We used NP-40S detergent for cell lysis that extracted the vast majority of CD4, CD8, and LCK molecules from thymocytes and lymph node (LN) cells (Figure S1A), excluding the possibility that our results are influenced by a potential insolubility of these proteins. We used an anti-CD8β antibody for pull down and anti-CD8α for detection to exclude eventual CD8αα homodimers that do not promote TCR signaling (Witte et al., 1999). For the pull down and detection of CD4, two non-competing antibody clones were used (Figures S1B–S1D). The concentration of the detection antibodies was titrated to use saturating concentrations (Figure S1E).

In agreement with the previous study (Stepanek et al., 2014), we observed a substantially higher frequency of LCK-coupled CD4 coreceptors than CD8 coreceptors in DP thymocytes (Figure 1A). Interestingly, the difference between CD4-LCK and CD8-LCK interactions was much less pronounced in mature peripheral T cells than in DP thymocytes (Figure 1A). Upon T cell maturation, the CD8-LCK binding stoichiometry increased ~13-fold, whereas the percentage of CD4 molecules coupled to LCK increased only ~2-fold (Figure 1A).

We addressed the relative changes of coreceptor-LCK binding stoichiometries between thymocytes and mature T cells by using an independent technique. We performed conventional immunoprecipitation from cell lauryl-maltoside lysates followed by immunoblotting (Figures 1B and 1C). We observed differences in the apparent molecular weight of CD8α between thymocytes and peripheral T cells (Figure 1B). First, the apparent molecular weight of CD8α was lower in thymocytes than in peripheral T cells. This shift is caused by more intensive sialylation of CD8 in peripheral cells than in thymocytes (Daniels et al., 2001; Merry et al., 2003, Moody et al., 2001). Accordingly, the removal of the sialic acid chains by neuraminidase normalized the apparent molecular weight of CD8α but did not substantially affect the relative intensities of the detecting antibodies in immunoblotting or flow cytometry (Figures S1F and S1G). Second, there was an additional lower band detected exclusively in the thymocytes (Figure 1B). This band corresponds to the truncated isoform CD8α' (Zamoyska et al., 1989; Zamoyska and Parnes, 1988). The analysis of the LCK to coreceptor ratio in this experiment showed that upon T cell maturation, the CD8-LCK binding stoichiometry increased ~9-fold, whereas the percentage of CD4 molecules coupled to LCK increased only ~2-fold. Thus, these results were in a good agreement with the FC-IP data.

The dramatic changes in CD8-LCK coupling frequency upon maturation is most likely caused by two factors. First, we and others observed that the truncated isoform CD8α' devoid of the LCK-binding site is present in thymocytes but not in peripheral T cells (Figure 1B). Interestingly, the expression of CD8α' is regulated post-transcriptionally, as there is almost no difference in the CD8α'-encoding RNA levels between the two T cell stages (Figure S1H) (Zamoyska and Parnes, 1988). However, CD8α' constitutes only ~30% of the total CD8α in thymocytes (Figure S1I), suggesting that there must be an additional mechanism for the dynamic regulation of CD8-LCK coupling. Because the vast majority of LCK molecules are coupled to CD4 or CD8 in DP thymocytes (Van Laethem et al., 2007) and because CD4 has been shown to have higher affinity to LCK than CD8 has *in vitro* (Kim et al., 2003), CD4 sequesters LCK from CD8 at the DP stage, which does not occur in mature CD8$^+$ T cells.

We previously developed the "LCK come&stay/signal duration model" to predict TCR signaling output by using a set of parameters including TCR density, antigen affinity, and coreceptor-LCK stoichiometry (Stepanek et al., 2014). The model is based on the kinetic proof-reading principle (McKeithan, 1995). It assumes that LCK recruitment and phosphorylation of the TCR/ZAP70 complex must be accomplished during the interaction of the TCR with the pMHC to trigger the TCR. The model assumes that the triggered TCR continuously transduces the signal downstream as long as it is occupied by the antigen. This model was the only one among a couple of constructed models that could explain the importance of the coreceptor-LCK binding in the antigen affinity discrimination in DP thymocytes, which was observed experimentally (Stepanek et al., 2014). We use this relatively simplistic model here to obtain testable predictions of how the dynamics of CD4-LCK and CD8-LCK coupling regulates the T cell responses to antigens. To assess how the differences

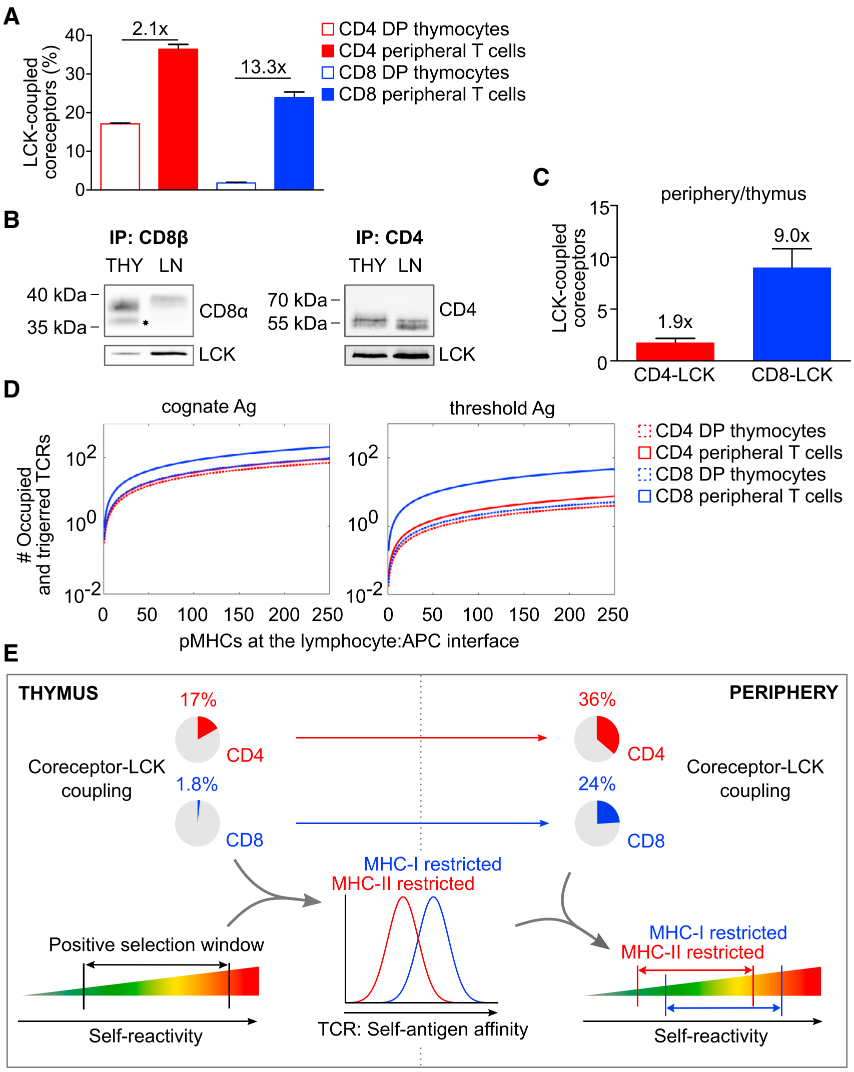

Figure 1. The Dynamics of the Coreceptor-LCK Coupling Predicts Self-Reactivity

(A) Mature T cells or DP thymocytes were lysed and incubated with beads coated with antibodies to CD4 (RM4-4) or CD8β (53-5.8). Beads were probed with PE-conjugated antibodies to LCK (3a5), CD8α (53-5.7), or CD4 (H129.19) and analyzed by flow cytometry. Calculated CD4-LCK or CD8-LCK stoichiometry for thymocytes and mature T cells is shown. Mean + SEM; n = 3–5 mice in 3–4 independent experiments.

(B and C) CD4 (H129.19) or CD8β (53-5.8) were immunoprecipitated from lysates of thymocytes or enriched peripheral CD8$^+$ and CD4$^+$ T cells from WT mice, followed by immunoblotting using anti-CD4 (D7D2Z), anti-CD8α (D4W2Z). and anti-LCK (3a5) antibodies. (B) Representative experiments for CD8β and CD4 immunoprecipitation. CD8α' truncated isoform is marked with an asterisk. (C) Ratio of LCK-coupled coreceptors (periphery/thymus). Mean + SEM; n = 3–5 samples (pooled 2–3 mice) in 3–4 independent experiments.

(D) TCR signal intensity predicted by the "LCK come&stay/signal duration model" (Stepanek et al., 2014) induced by strong cognate or suboptimal antigens (at the threshold for negative selection) in MHCI- or MHCII-restricted DP or mature T cells. The TCR signal intensity corresponds to the number of signaling TCRs and is shown as a function of antigen density. The input data correspond to the parameters obtained from monoclonal OT-I and B3K508 T cells (Stepanek et al., 2014).

(E) Schematic illustration of the prediction of the mathematical model applied to the process of T cell selection. The coreceptor-LCK coupling in the thymocytes sets the self-antigen affinity window of the positively selected T cell, resulting in higher affinity to self-antigens in the MHCI-restricted than in the MHCII-restricted T cells. Increased CD8-LCK, but not CD4-LCK, coupling frequency in mature T cells leads to the increased sensitivity of peripheral CD8 T cells to suboptimal antigens. Altogether, mature CD8$^+$ T cells have, on average, higher level of self-reactivity than CD4$^+$ T cells.

See also Figure S1, Table S1, and Data S1.

in the dynamics of CD4-LCK and CD8-LCK coupling influences the TCR signaling, we used our experimental CD4- and CD8-LCK stoichiometry data as well as the quantification of the percentage of phosphorylated LCK molecules, and the TCR levels on mature CD4$^+$ and CD8$^+$ T cells (Figures S1J–S1M, Table S1) as inputs for the LCK come&stay/signal duration model.

The model predicts that MHCI- and MHCII-restricted T cells and DP thymocytes exhibit comparable responses to their high-affinity cognate antigens (Figure 1D). However, the stoichiometry of the coreceptor-LCK interaction was shown to be limiting, specifically for signaling induced by suboptimal antigens (Erman et al., 2006; Stepanek et al., 2014; Drobek et al., 2018). We took advantage of the fact that the affinities to self-antigens at the threshold for negative selection are known for both MHCI-restricted and MHCII-restricted thymocytes (Daniels et al., 2006; Naeher et al., 2007; Stepanek et al., 2014), and we used these parameters in the mathematical model. The model predicts that partial-negative-selecting antigens induce stronger TCR signaling in CD8$^+$ mature peripheral T cells than in peripheral CD4$^+$ T cells or in MHCI- and MHCII-restricted DP thymocytes (Figure 1D). These results suggest that peripheral MHCI-restricted CD8$^+$ T cells, but not MHCII-restricted CD4$^+$ T cells, could be activated by positive selecting or only partial negative selecting self-antigens.

CD8$^+$ T Cells Are More Reactive to Suboptimal Antigens Than CD4$^+$ T Cells *Ex Vivo*

Based on the dynamics of the CD4- and CD8-LCK binding stoichiometry and the predictions of the mathematical model, we hypothesize that MHCI-restricted T cells, but not MHCII-restricted T cells, increase their sensitivity to suboptimal antigens upon maturation. We reasoned that CD8$^+$ T cells are, therefore, on average more self-reactive than CD4$^+$ T cells (Figure 1E). In the next steps, we addressed our hypothesis experimentally.

We used monoclonal mature T cells and thymocytes from *Rag2*-deficient mice bearing either MHCI-restricted

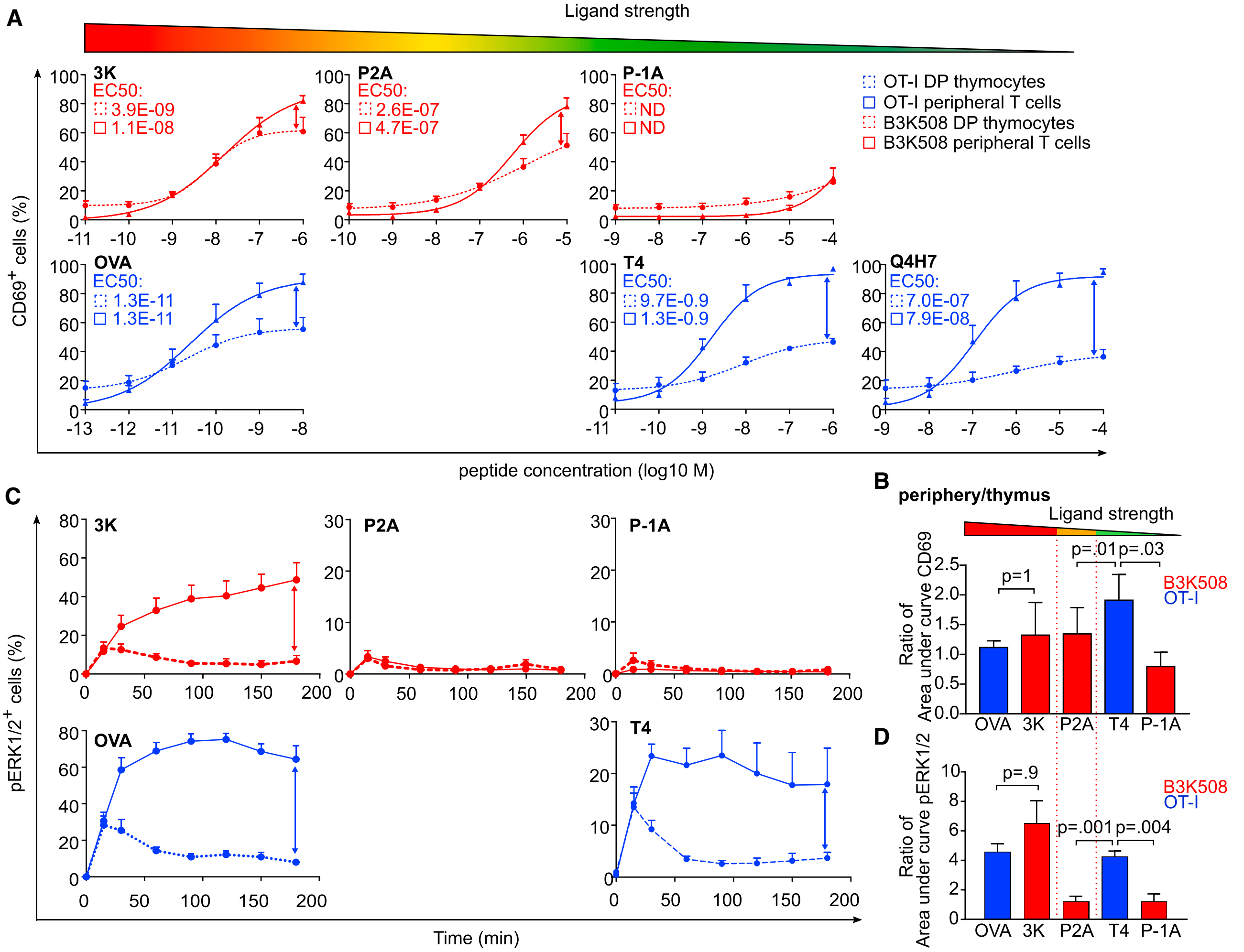

Figure 2. CD8$^+$ T Cells Are More Sensitive to Suboptimal Antigens Than CD4$^+$ T Cells *In Vitro*

LN T cells or thymocytes from OT-I mice (blue) or B3K508 mice (red) were stimulated by BMDCs loaded with indicated concentrations of indicated peptides overnight.

(A and B) CD69 levels on T cells were analyzed by flow cytometry. EC_{50} concentrations of peptides are indicated (A). The ratio of the area under curve of the % CD69$^+$ T cells in the periphery versus thymus (B). Mean + SEM; n = 4 mice in 4 independent experiments.

(C and D) The cells were stimulated by BMDCs loaded with a 10^{-5}-M concentration of indicated peptides, fixed at indicated time points, and analyzed for phosphorylation of ERK1/2 by flow cytometry. Mean + SEM; n = 6–10 independent experiments (C). The ratio of area under curve of % pERK1/2$^+$ T cells in the periphery versus thymus. Mean + SEM, n = 6–10 mice in 6–10 independent experiments (D). Statistical analysis was performed using 2-tailed Mann-Whitney test.

See also Figure S2.

OT-I-transgenic TCR (specific to H-2K^b-SIINFEKL; OVA) or MHCII-restricted B3K508-transgenic TCR (specific to H-2A^b-FEAQKAKANKAKAVD; 3K) as our experimental model. The advantage of these monoclonal models is that there is a wide range of well-characterized cognate-altered peptide ligands covering negative selectors, partial negative selectors, and positive selectors (Daniels et al., 2006; Huseby et al., 2006; Keck et al., 2014; Stepanek et al., 2014; Figure S2A). TCR expression is higher in B3K508 T cells than in OT-I T cells, mimicking the situation in polyclonal T cells (Figures S1M and S2B).

Upon overnight stimulation with bone-marrow-derived dendritic cells (BMDCs) pulsed with the cognate peptides or their lower affinity variants, we measured the expression of an activation marker, CD69, in the monoclonal T cells and thymocytes (Figures 2A, 2B, S2C, and S2D). We compared CD69 upregulation in mature T cells and DP thymocytes by calculating the ratio of the corresponding areas under curve for each antigen (Figure 2B) or the ratio of the maximal response (Figure S2D). The responses of OT-I and B3K508 mature T cells and DP thymocytes to the high-affinity antigens (OVA and 3K, respectively) were comparable. However, the mature OT-I T cells exhibited stronger responses than DP thymocytes when stimulated with suboptimal antigens T4 and Q4H7, a partial negative selector and a positive selector, respectively (Figures 2A, 2B, S2C, and S2D). In the case of B3K508 mice, the mature T cells and DP thymocytes showed comparable responses to suboptimal antigens P2A and P-1A, a relatively weak negative selector and a partial negative selector, respectively (Figures 2A, 2B, S2C, and S2D).

OPEN
ACCESS
CellPress

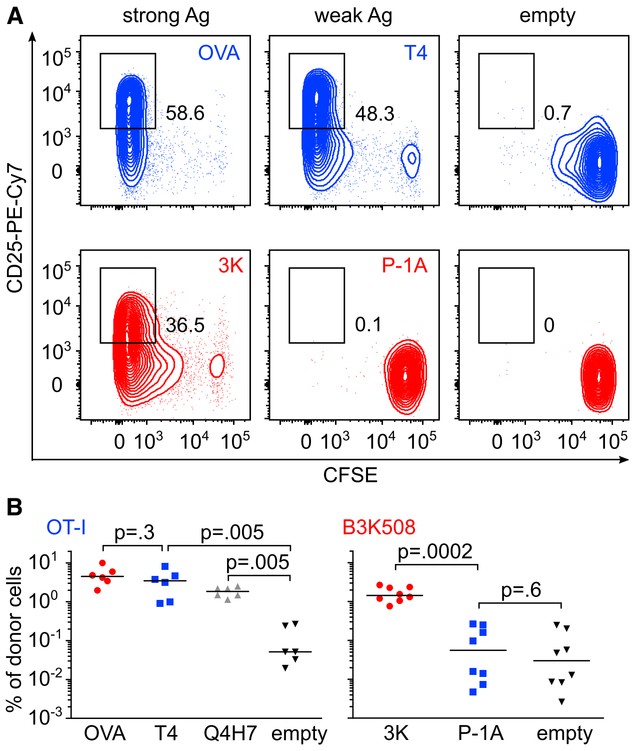

Figure 3. CD8+ T Cells Are More Sensitive to Suboptimal Antigens Than CD4+ T Cells *In Vivo*

CFSE-loaded LN cells from OT-I mice and B3K508 mice were injected into congenic Ly5.1 WT mice. The mice were infected with transgenic *Lm* expressing indicated peptides. Four days after the infection, viable splenic donor T cells (gated as CD3+ CD4+ Va2+ Ly5.2+ for B3K508 T cells and CD3+ CD8+ Va2+ Ly5.2+ for OT-I T cells) were analyzed for proliferation (CFSE) and CD25 expression by flow cytometry.

(A) Representative animals out of 6–8 per group.

(B) The percentage of donor cells among all splenic CD4+ or CD8+ T cells is shown. n = 6–8 mice in 4 independent experiments. Statistical analysis was performed using 2-tailed Mann-Whitney test.

See also Figure S3.

In a next step, we studied a proximal TCR signaling event, a phosphorylation of kinases ERK1 and ERK2 (Figures 2C, 2D and S2E–S2G). As the mature T cells and DP thymocytes had distinct kinetics of ERK1/2 phosphorylation, we used the overall response calculated as the area under curve (Figure 2D) or maximal response (Figure S2G) for quantification. We calculated the mature T cell/DP thymocytes ratio for each antigen. In the case of OT-I T cells, mature T cells showed an ∼4.5-fold stronger overall response than DP thymocytes to both the high-affinity antigen OVA and the suboptimal antigen T4 (Figures 2C and 2D). However, B3K508 mature T cells showed a substantially stronger response to the high-affinity antigen than DP thymocytes (∼6.5-fold), but the responses of B3K508 mature T cells and DP thymocytes to suboptimal antigens P2A and P-1A were comparable (Figures 2C and 2D).

Overall, CD69 upregulation and pERK1/2-phosphorylation were in line with the mathematical model predicting that the responses to suboptimal antigens are augmented upon maturation of MHCI-restricted, but not MHCII-restricted, T cells. However, it should be noted here that the responses of B3K508 T cells to P2A (pERK1/2) and P-1A (both pERK1/2 and CD69) were weak, which limits our conclusions.

CD8+ T Cells Are More Reactive to Suboptimal Antigens Than CD4+ T Cells *In Vivo*

We next examined the activation of T cells *in vivo*. In this assay, we took advantage of the fact that the ability of particular antigens to induce negative selection in OT-I and B3K508 thymocytes has been established previously (Daniels et al., 2006; Huseby et al., 2006; Keck et al., 2014; Stepanek et al., 2014; Wyss et al., 2016). Thus, we could monitor T cell responses to high-affinity cognate antigens and partial-negative-selecting antigens in the periphery. We transferred CFSE-labeled OT-I and B3K508 peripheral T cells into congenic Ly5.1 mice. Subsequently, we infected the mice with *Listeria monocytogenes* (*Lm*) expressing the cognate antigens for the transferred T cells. We analyzed the expansion, proliferation, and CD25 upregulation in the donor T cells 4 days later. Both OT-I and B3K508 T cells exhibited strong proliferation, expansion, and CD25 upregulation upon infection, with *Lm* carrying the respective high-affinity cognate antigens (OVA and 3K) (Figures 3A and 3B; Figures S3A–S3D). In the case of OT-I T cells, *Lm* carrying the partial-negative-selecting antigen T4 or even a positive-selecting antigen Q4H7 induced substantial expansion, proliferation, and CD25 upregulation, whereas non-cognate empty *Lm* did not induce a detectable response (Figures 3A and 3B; Figures S3A and S3B). In striking contrast to OT-I T cells, B3K508 T cells did not respond to *Lm* expressing the partial-negative-selecting antigen P-1A (Figures 3A and 3B; Figures S3A and S3B). Collectively, these data reveal that peripheral CD8+ T cells show a robust *in vivo* response to antigens with low affinity as partial negative selectors or even positive selectors, whereas peripheral CD4+ T cells are not able to respond to partial-negative-selecting antigens at all.

CD8+ T Cells Experience Stronger Homeostatic TCR Signals Than CD4+ T Cells

The results of *in vitro* and *in vivo* assays using monoclonal MHCI- and MHCII-restricted T cells corresponded well to the predictions of the mathematical model. If we translate these findings to the polyclonal repertoire, we can hypothesize that the CD8+ T cell population is, on average, more self-reactive than the CD4+ population because only the CD8+ subset contains T cells that are able to respond to the positive- and partial-negative-selecting self-antigens at the periphery.

The self-reactivity of peripheral T cells determines the intensity of homeostatic signaling at the basal state. We generated and analyzed LCK-deficient mice to (1) validate our tools for the detection of proximal signaling intermediates of tonic signaling by using phospho-specific antibodies by flow cytometry and (2) to address the role of LCK in the homeostatic TCR signaling. The $Lck^{-/-}$ thymocytes showed partial blocks in the β selection and positive selection (Figure S4A), as previously reported (Molina et al., 1992). Reduced LCK levels in heterozygous $Lck^{+/-}$ and in $Lck^{-/-}$ mice lead to a gradual decrease in TCRζ and ZAP70 phosphorylation and overall tyrosine phosphorylation in both CD4 and CD8 peripheral T cells (Figure 4A). These results

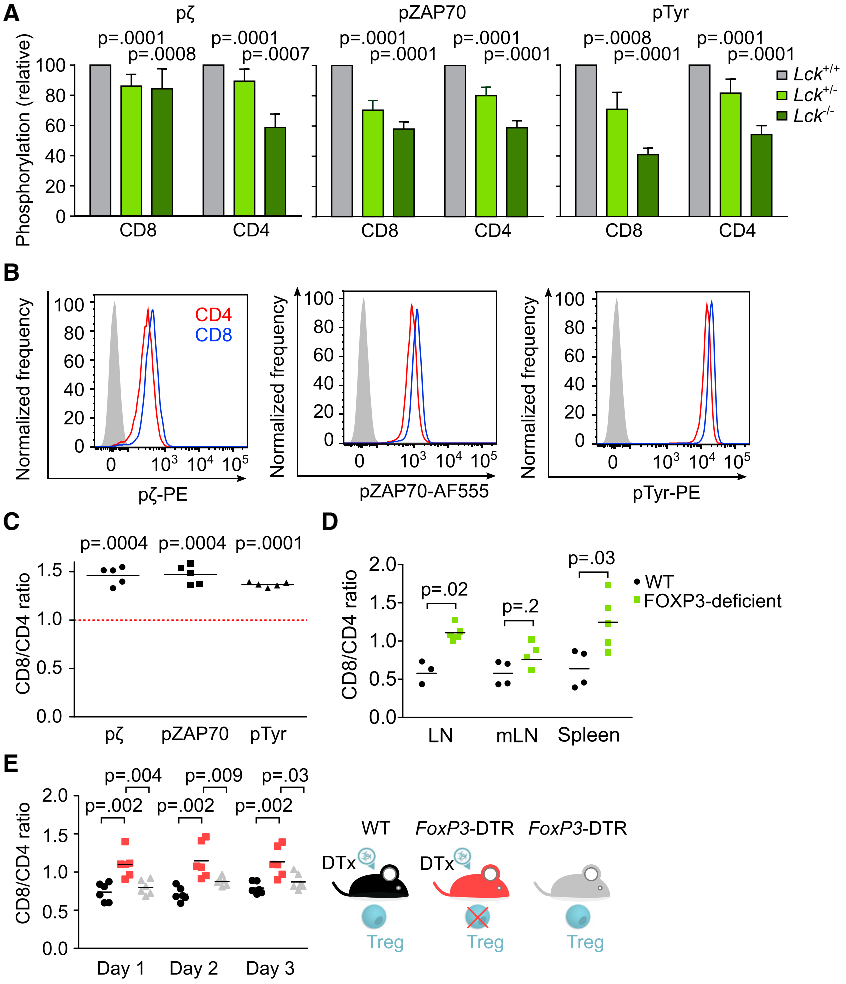

Figure 4. Polyclonal CD8$^+$ T Cells Show Stronger Homeostatic TCR Signaling Than CD4$^+$ T Cells

(A–C) Fixed and permeabilized LN T cells from WT mice were stained with antibodies to TCRβ, CD4, CD8, pTCRζ chain, pZAP70, and overall tyrosine phosphorylation and analyzed by flow cytometry. Comparison of basal signaling in CD8$^+$ CD44$^-$ and CD4$^+$ CD44$^-$ T cells from *Lck*$^{+/+}$, *Lck*$^{+/-}$, and *Lck*$^{-/-}$ mice. Phosphorylation level (geometric mean fluorescence intensity [gMFI]) relative to *Lck*$^{+/+}$ mice is shown. Mean + SEM; n = 7 mice in 7 independent experiments. Statistical analysis was performed by one sample t test (hypothetical mean value = 1) (A). Comparison of basal signaling in CD4$^+$ and CD8$^+$ T cells. A representative experiment out of 5 independent experiments in total (B). Ratio of phosphorylation levels (gMFI) of CD8$^+$ versus CD4$^+$ peripheral T cells in pTCRζ, pZAP70, and overall tyrosine phosphorylation for each mouse. Mean; n = 5 mice in 4 independent experiments. Statistical analysis was performed by one sample t test (hypothetical mean value = 1) (C).

(D) The LN, mLN, and splenic T cells of *Foxp3*-deficient mice and their WT littermates were analyzed by flow cytometry. The ratio of CD8$^+$ to CD4$^+$ T cells is shown. Mean; n = 4–5 mice from 2 experiments. Statistical analysis was performed by 2-tailed Mann-Whitney test.

(E) The peripheral LN T cells from *Foxp3*-DTR mice after the administration of diptheria toxin, untreated *Foxp3*-DTR mice, and WT mice after the administration of diptheria toxin were analyzed. The ratio of CD8$^+$ to CD4$^+$ T cells is shown. n = 6 mice in 3 independent experiments; mean. Statistical analysis was performed by 2-tailed Mann-Whitney test.

See also Figure S4.

show that LCK is a major factor regulating the strength of the homeostatic TCR signaling in resting peripheral T cells, which is in line with our model.

We examined the intensity of homeostatic TCR signaling to compare the level of self-reactivity of polyclonal peripheral CD4$^+$ and CD8$^+$ T cells. Higher levels of TCRζ and ZAP70 phosphorylation and overall tyrosine phosphorylation in CD8$^+$ T cells than in CD4$^+$ T cells suggested that CD8$^+$ T cells receive stronger homeostatic signals from self-antigens than CD4$^+$ T cells (Figures 4B and 4C). These observations were not substantially influenced by the inclusion of CD4$^+$ regulatory T cells (Tregs), as the comparison of CD8$^+$ T cells to conventional FOXP3$^-$ CD4$^+$ T cells showed similar results (Figures S4B and S4C). Moreover, the higher intensity of basal TCR signaling in CD8$^+$ T cells than in CD4$^+$ T cells was not caused by higher surface TCR levels in CD8$^+$ T cells. On the contrary, CD8$^+$ T cells have a lower surface TCR expression than CD4$^+$ T cells (Figure S1M). Overall, these data supported the hypothesis that CD8$^+$ T cells are more self-reactive than CD4$^+$ T cells.

The higher level of self-reactivity of CD8$^+$ T cells than of CD4$^+$ T cells indicates that CD8$^+$ T cells might be more susceptible to hyperproliferation than CD4$^+$ T cells. To address this hypothesis, we used Treg deficiency as a model for a systemic breakdown of peripheral tolerance. We observed that the ratio of CD8$^+$/CD4$^+$ T cells is significantly higher in FOXP3-deficient 2- to 3-week-old mice devoid of Tregs than in the healthy littermates (Figure 4D). In a complementary assay, we observed the effect of acute depletion of Tregs by injecting diphtheria toxin (DT) into *Foxp3*-DTR mice, expressing the DT receptor in FOXP3$^+$ T cells (Kim et al., 2007). The CD8$^+$/CD4$^+$ T cell ratio significantly increased in adult Treg-depleted mice compared to untreated *Fox3*-DTR mice and to wild-type (WT) mice injected with the DT (Figure 4E). These results are consistent with the hypothesis that the polyclonal CD8$^+$ T cell population receives stronger signals from self-antigens than the CD4$^+$ T cell population and that CD8$^+$ T cells are more prone to hyperproliferation than CD4$^+$ T cells when the Treg-mediated tolerance fails. However, we cannot exclude that additional differences between CD8$^+$ versus CD4$^+$ (e.g., differential expression of cytokine receptors) play a role in this assay.

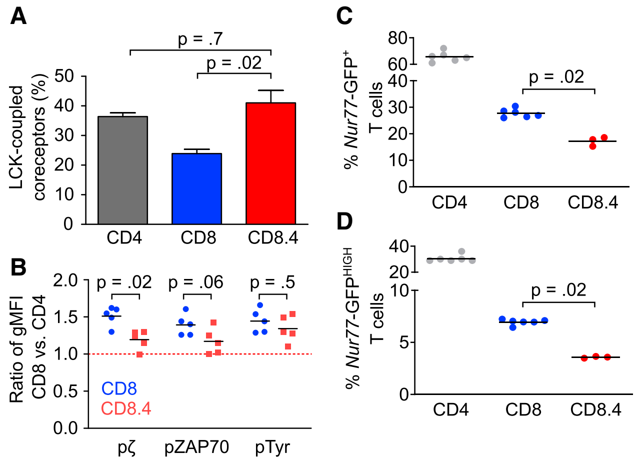

Figure 5. Role of Coreceptor-LCK Coupling in Self-Reactivity of T Cell Subpopulations

(A) CD4-LCK, CD8-LCK, or CD8.4-LCK stoichiometry in LN T cells of WT and CD8.4 mice was analyzed. Mean + SEM; n = 4–9 mice from 4–8 independent experiments. Statistical analysis was performed by 2-tailed Mann-Whitney test. The CD4-LCK and CD8-LCK stoichiometries are the same as that shown in Figure 1A.

(B) Ratio of MFI levels of pTCRζ, pZAP70, and overall tyrosine phosphorylation in CD8$^+$ versus CD4$^+$ peripheral T cells in WT and CD8.4 chimeric mice is shown. Mean, n = 5 mice in 3 independent experiments. Statistical analysis was performed by 2-tailed Mann-Whitney test. See Figure S5D for similar data.

(C and D) LN T cells from *Nur77*-GFP reporter mice were analyzed by flow cytometry. The CD8$^+$ CD44$^-$ CD62L$^+$ or CD8.4$^+$ CD44$^-$ CD62L$^+$ T cells were analyzed for the expression of the *Nur77*-GFP. The percentage of *Nur77*-GFP$^+$ and *Nur77*-GFP^{HIGH} cells is shown. Mean; n = 3–6 mice in 3–6 independent experiments. Statistical analysis was performed by 2-tailed Mann-Whitney test. See also Figure S5.

The Self-Reactivity of CD8$^+$ T Cells Is Regulated by the Dynamics of the CD8-LCK Coupling

The previous experiments and the prediction of the mathematical model show substantial differences between CD8$^+$ and CD4$^+$ T cells in terms of their response to suboptimal antigens and the basal signaling. In a next step, we addressed the link between the coreceptor-LCK coupling and the self-reactivity of mature T cells directly. We took advantage of a previously developed CD8.4 knockin mouse model (Drobek et al., 2018; Erman et al., 2006; Stepanek et al., 2014). In this mouse, MHCI-restricted T cells express a chimeric CD8.4 coreceptor that has the intracellular domain of CD8α replaced by the intracellular domain of the CD4 coreceptor. The coupling of CD8.4 coreceptor to LCK is comparable to CD4 both in the thymus and at the periphery (Stepanek et al., 2014) (Figure 5A). The basal phosphorylation of TCRζ in CD8.4 T cells was significantly lower than in CD8$^+$ T cells, and the difference in the ZAP70 phosphorylation was close to significant (Figure 5B), indicating that peripheral CD8$^+$ T cells are, on average, more self-reactive than CD8.4$^+$ T cells. We obtained similar results when we repeated this key experiment in a different animal facility (Figure S5D). Because the phosphorylation of ZAP70 and TCRζ and overall tyrosine phosphorylation were only slightly increased in CD8.4$^+$ compared with CD4$^+$ T cells, we concluded that the changes in coreceptor-LCK stoichiometry during development are a major cause of the differences in basal TCR signaling between CD8$^+$ and CD4$^+$ T cells.

Expression of an orphan nuclear receptor, *Nur77*, is very sensitive even to weak TCR signaling. For this reason, the *Nur77*-GFP reporter mouse has been used to study TCR signals induced by self-antigens (Moran et al., 2011). The *Nur77*-GFP signal is stronger in CD4$^+$ than in CD8$^+$ T cells (Moran et al., 2011) (Figures S5A and S5B), suggesting that CD4$^+$ T cells might have stronger TCR signaling than CD8$^+$ T cells. Because these results are contradictory to our analysis of basal TCR signaling (Figures 4B and 4C), we addressed it in a greater detail. We compared GFP expression in CD8$^+$ *Nur77*-GFP and CD8.4$^+$ *Nur77*-GFP T cells. We observed a significantly higher frequency of GFP$^+$ and GFP^{HIGH} cells among CD8$^+$ than among CD8.4$^+$ T cells (Figures 5C and 5D; Figure S5C), supporting our previous data that the dynamics of the coreceptor-LCK stoichiometry sets the level of T cell self-reactivity. In the light of these data, we suggest that the previously reported higher expression of *Nur77* in CD4$^+$ T cells than in CD8$^+$ T cells reflects a differential regulation of this gene in these two very different T cell types rather than the differences in basal TCR signaling per se.

The sizes of CD4, CD8, and CD8.4 naive T cells are comparable in terms of cellular dry mass (Figure S5E) and forward scatter signal (Figure S5F). The TCR levels are the highest in CD4 T cells and comparable in CD8 and CD8.4 T cells (Figures S1M and S5G). The expression level of ZAP70 is comparable among these cell types (Figure S5H). Thus, we could exclude the possibility that the cell size variation or TCR or ZAP70 expression is responsible for the observed differences in the homeostatic TCR signaling.

Altogether, our data indicate that the stoichiometry of the CD4- and CD8-LCK interactions and their changes during T cell development establish the level of self-reactivity of mature T cells. The dynamic regulation of the CD8-LCK stoichiometry causes CD8$^+$ T cells to be, on average, more self-reactive than CD4$^+$ T cells.

DISCUSSION

Our results show that the developmental dynamics of CD4-LCK and CD8-LCK stoichiometries differ substantially in mice. A relatively high number of CD4 molecules are coupled to LCK in developing DP thymocytes (Stepanek et al., 2014), and this coupling is only mildly increased during the maturation of CD4$^+$ T cells. In contrast, CD8-LCK stoichiometry is relatively low in DP thymocytes (Stepanek et al., 2014) when positive selection and most of the negative selection occurs. The number of CD8 molecules carrying LCK dramatically increases upon maturation. This is partially caused by the expression of the truncated CD8α' variant (Zamoyska et al., 1989). CD8α' is expressed only on the surface of thymocytes but not of mature T cells (Zamoyska and Parnes, 1988). However, the major mechanism causing the low CD8-LCK coupling in DP thymocytes is the preferential sequestering of LCK (Van Laethem et al., 2007) by CD4, which has a higher affinity for LCK than CD8 (Kim et al., 2003).

The stoichiometry between the coreceptors and LCK is a limiting factor for triggering the TCR signaling by suboptimal antigens (Stepanek et al., 2014). Our mathematical model (Stepanek et al., 2014) made two interesting predictions: (1) mature

peripheral CD8$^+$ T cells, but not CD4$^+$ T cells, are more sensitive to suboptimal cognate antigens than DP thymocytes with the same specificity and (2) mature CD8$^+$ T cells are more self-reactive than mature CD4$^+$ T cells. Our experimental data using well-defined monoclonal MHCI- and MHCII-restricted T cells as well as polyclonal murine T cells supported the predictions of the model. The experiments using T cells expressing the chimeric CD8.4 coreceptor showed that the higher self-reactivity of CD8$^+$ T cells compared to CD4$^+$ T cells is at least partially caused by the differential developmental kinetics of the CD8-LCK versus CD4-LCK stoichiometries.

One of the key mechanisms of self-tolerance is the removal of overtly self-reactive T cell clones during negative selection in the thymus. However, the self-reactivity of positively selected mature T cells is beneficial for their survival and immune responses (Mandl et al., 2013; Fulton et al., 2015; Swee et al., 2016; Stefanová et al., 2002). These two counteracting mechanisms generate a pressure for an optimal level of self-reactivity of mature T cells. Because the effector roles of CD4$^+$ and CD8$^+$ T cells in the immune responses are very different, it is very likely that the optimal level of self-reactivity differs between these two populations. However, the proximal TCR signaling pathway is identical in CD8$^+$ and CD4$^+$ T cells, which limits the possibilities of tuning the optimal level of self-reactivity for CD4$^+$ and CD8$^+$ T cells individually by evolutionary processes. The coreceptors represent the exceptional part of the TCR signaling machinery that differs between CD4$^+$ and CD8$^+$ T cells, and thus, it represents the ideal target for the evolutionary tuning of optimal self-reactivity of CD4$^+$ and CD8$^+$ T cells separately. The increase in CD8-LCK, but not CD4-LCK, stoichiometry during the maturation contributes to setting the higher level of self-reactivity of mature CD8$^+$ T cells than of mature CD4$^+$ T cells. Because CD4$^+$ helper T cells modulate immune responses by acting on many other leukocytes, an autoimmune response of a CD4$^+$ T cell might be more harmful than an autoimmune response of a CD8$^+$ T cell. This might explain why we observed a buffering gap between the self-antigen affinity required for negative selection and affinity required for inducing an immune response in CD4$^+$ T cells. In contrast, CD8$^+$ T cells are able to induce a robust response to partial negative selectors and even to positive selectors. In the case of CD8$^+$ T cells, the benefit from having a higher level of self-reactivity might overcome the risk of inducing autoimmunity.

Our findings were surprising because previous studies often suggested the opposite, i.e., that mature peripheral T cells are less sensitive to antigens than DP thymocytes (Ebert et al., 2009; Li et al., 2007; Lucas et al., 1999) and that peripheral CD4$^+$ T cells are more self-reactive than CD8$^+$ T cells (Moran et al., 2011).

Our data highlight the role of CD8-LCK stoichiometry as the main cause of the increase in the responsiveness to suboptimal antigens, including self-antigens, upon maturation of CD8$^+$ T cells. Moreover, pre-selection DP thymocytes exhibit substantially lower levels of surface TCR than mature T cells, which might also contribute to their lower sensitivity. In contrast, multiple mechanisms selectively enhancing the responses of thymocytes or suppressing the signaling in mature T cells were proposed (reviewed in Gaud et al., 2018). These mechanisms include sialyation of CD8 in peripheral T cells (Starr et al., 2003) and higher expression of positive regulators of TCR signaling, a voltage-gated sodium channel (Lo et al., 2012), Themis (Choi et al., 2017; Fu et al., 2009; Johnson et al., 2009), TESPA1 (Wang et al., 2012; Liang et al., 2017), and miRNA-181a (Li et al., 2007; Ebert et al., 2009) in thymocytes than in mature T cells.

Because our data concerning MHCII-restricted T cells do not show substantial differences between the immature and mature T cells, they are not in dramatic contrast to previous studies (Ebert et al., 2009; Li et al., 2007; Lucas et al., 1999). However, our data showing that CD8$^+$ T cells increase their sensitivity to suboptimal antigens upon maturation are contradictory to some previous reports (Davey et al., 1998; Starr et al., 2003). Most likely, differences in the experimental *ex vivo* protocols caused the discrepancy. For instance, the expression of costimulatory and inhibitory ligands on the APCs might selectively regulate responses of thymocytes and/or mature T cells. Thus, the usage of different cells as APCs could be the source of the inconsistencies among different studies. For this and other reasons, we believe that the *in vivo* data are more relevant than the *ex vivo* experiments. We showed that partial-negative-selecting and positive-selecting antigens are able to trigger a significant activation of CD8$^+$, but not CD4$^+$ T cells, *in vivo*. These experiments are in a very good agreement with previously published *in vivo* studies (Keck et al., 2014; King et al., 2012; Koehli et al., 2014; Zehn et al., 2009; Enouz et al., 2012), although a side-by-side comparison of CD8$^+$ and CD4$^+$ T cells has not been carried out previously.

The higher self-reactivity of CD4$^+$ T cells than CD8$^+$ T cells has been suggested based on the higher expression of *Nur77*-GFP and CD5 in CD4$^+$ T cells than in CD8$^+$ T cells (Moran et al., 2011; Hogquist and Jameson, 2014). However, it is possible that the transcription of these reporter genes is regulated in a cell-type-specific manner (Moran et al., 2011). In such scenario, the markers are not reliable for a comparison between different T cell subsets. To avoid comparing apples and oranges, we examined CD8$^+$ T cells and CD8.4$^+$ T cells expressing a chimeric coreceptor, recapitulating the dynamics of CD4-LCK, but selecting MHCI-restricted T cells. CD8.4$^+$ T cells showed lower basal expression of *Nur77*-GFP than CD8$^+$ T cells. These results indicate that the changes in the coreceptor-LCK stoichiometry during maturation determine the self-reactivity of T cells. Moreover, the data suggest that the *Nur77*-GFP reporter is most likely differentially regulated in CD8$^+$ and CD4$^+$ T cells. To avoid artifacts caused by unique gene expression programs in CD4$^+$ and CD8$^+$ T cells, we focused on the basal TCR-dependent phosphorylation of TCRζ and ZAP70. We believe that this analysis of the proximal TCR signaling is the most reliable approach for the comparison of basal TCR signaling among different T cell subsets. It should be noted here that we cannot formally exclude that, besides LCK, other potential interacting partners of CD4 and/or CD8 might contribute to the differences between the CD8WT$^+$ and CD8.4$^+$ T cells.

Data about the coreceptor-LCK coupling in human immature and mature T cells are not available. Moreover, the level of self-reactivity of human CD4$^+$ and CD8$^+$ T cells has not been addressed. These parameters can be very different from mice.

However, the principle of tuning the self-reactivity by the regulation of coreceptor-LCK stoichiometry could be very general because it represents an ideal target to set up the optimal self-reactivity of $CD4^{+}$ and $CD8^{+}$ T cells separately. Subsequent work should focus on the link between self-reactivity and coreceptor-LCK stoichiometry in human T cells.

Mice with point mutations disabling the CD8-LCK and/or CD4-LCK interactions would be required for a thorough understanding of the role of the coreceptor-LCK interaction in the T cell biology. However, our data suggest that targeting the CD4- and CD8-LCK interactions might reduce T cell self-reactivity. Thus, these interactions represent a potential target for the therapy of autoimmune diseases.

STAR★METHODS

Detailed methods are provided in the online version of this paper and include the following:

- KEY RESOURCES TABLE
- LEAD CONTACT AND MATERIALS AVAILABILITY
- EXPERIMENTAL MODEL AND SUBJECT DETAILS
  - Mice
  - Bone marrow-derived dendritic cells (BMDC)
- METHOD DETAILS
  - Flow cytometric immunoprecipitation assay
  - Analysis of soluble and insoluble fractions
  - Immunoprecipitation of surface coreceptors
  - Neuraminidase treatment
  - Quatitative PCR
  - Mathematical model
  - T cell enrichment
  - Flow cytometry analysis and sorting
  - Determination of LCK phosphorylation status
  - Determination of TCR and coreceptor levels
  - Antigen presentation assay
  - Listeria infection
  - Quantitative phase imaging
  - Antibody competition binding assay
- QUANTIFICATION AND STATISTICAL ANALYSIS
- DATA AND CODE AVAILABILITY

SUPPLEMENTAL INFORMATION

Supplemental Information can be found online at https://doi.org/10.1016/j.celrep.2020.01.008.

ACKNOWLEDGMENTS

We thank Ladislav Cupak, Barbara Hausmann, and Rosmarie Lang for their technical assistance and genotyping of mice. We thank Light Microscopy and Flow Cytometry at Institute of Molecular Genetics, namely, Zdenek Cimburek and Dr. Matyas Sima for cell sorting and Dr. Ivan Novotny for assistance with microscopy experiments. We thank Dr. Inken Beck and Dr. Petr Kasparek from the Czech Centre of Phenogenomics for generating $Lck^{-/-}$ mice. We thank Prof. Ed Palmer for his multisided support to the project. We thank Prof. Alfred Signer for providing us with CD8.4 mice. We thank Dr. Tomas Brdicka for providing us with hybridoma-produced antibodies (anti-CD4, anti-CD11b). We thank Dr. Peter Draber for comments on the manuscript. This project was supported by the Swiss National Science Foundation (Promys, IZ11Z0_166538), the Czech Science Foundation (GJ19-03435Y), ERC Starting Grant (FunDiT) to O.S., and ERC Starting Grant (ProtecTC) to D.Z. L.W. is supported by SNSF Early Postdoc Mobility Scholarship (P2BSP3_168719). C.G. is supported by Novartis Foundation. The Group of Adaptive Immunity at the Institute of Molecular Genetics in Prague is supported by an EMBO Installation grant, the Institute of Molecular Genetics of the Czech Academy of Sciences (RVO 68378050), and the J.E.Purkyne Fellowship by the Czech Academy of Sciences (to O.S.). V.H. is a student of the Faculty of Science, Charles University, Prague. The animal facility of the IMG is a part of the Czech Centre for Phenogenomics, and the work there was supported, in part, by grants LM2015040, OP RDI CZ.1.05/2.1.00/19.0395, and OP RDI BIOCEV CZ.1.05/1.1.00/02.0109 provided by the Czech Ministry of Education, Youth and Sports and the European Regional Development Fund. Quantitative phase microscopy was performed in the Microscopy Centre - Light Microscopy Core Facility, IMG ASCR, Prague, Czech Republic, supported by grants (Czech-Bioimaging, MEYS LM2015062), "Centre of Model Organisms" OPPK (CZ.2.16/3.1.00/21547), and "Biomodels for health" (LO1419).

AUTHOR CONTRIBUTIONS

V.H. and O.S. planned experiments. V.H., A.D., D.M., C.G., L.W., V.N., and O.S. performed and analyzed experiments. C.G.K. and D.Z. contributed with unique biological material. O.S. performed the mathematical calculations. V.H. and O.S. wrote the manuscript. All authors reviewed the manuscript. O.S. conceived the study.

DECLARATION OF INTERESTS

The authors declare no competing interests.

Received: December 18, 2018
Revised: May 31, 2019
Accepted: January 2, 2020
Published: February 4, 2020

REFERENCES

Artyomov, M.N., Lis, M., Devadas, S., Davis, M.M., and Chakraborty, A.K. (2010). CD4 and CD8 binding to MHC molecules primarily acts to enhance Lck delivery. Proc. Natl. Acad. Sci. USA *107*, 16916–16921.

Barber, E.K., Dasgupta, J.D., Schlossman, S.F., Trevillyan, J.M., and Rudd, C.E. (1989). The CD4 and CD8 antigens are coupled to a protein-tyrosine kinase (p56lck) that phosphorylates the CD3 complex. Proc. Natl. Acad. Sci. USA *86*, 3277–3281.

Choi, S., Warzecha, C., Zvezdova, E., Lee, J., Argenty, J., Lesourne, R., Aravind, L., and Love, P.E. (2017). THEMIS enhances TCR signaling and enables positive selection by selective inhibition of the phosphatase SHP-1. Nat. Immunol. *18*, 433–441.

Daniels, M.A., Devine, L., Miller, J.D., Moser, J.M., Lukacher, A.E., Altman, J.D., Kavathas, P., Hogquist, K.A., and Jameson, S.C. (2001). CD8 binding to MHC class I molecules is influenced by T cell maturation and glycosylation. Immunity *15*, 1051–1061.

Daniels, M.A., Teixeiro, E., Gill, J., Hausmann, B., Roubaty, D., Holmberg, K., Werlen, G., Holländer, G.A., Gascoigne, N.R.J., and Palmer, E. (2006). Thymic selection threshold defined by compartmentalization of Ras/MAPK signalling. Nature *444*, 724–729.

Davey, G.M., Schober, S.L., Endrizzi, B.T., Dutcher, A.K., Jameson, S.C., and Hogquist, K.A. (1998). Preselection thymocytes are more sensitive to T cell receptor stimulation than mature T cells. J. Exp. Med. *188*, 1867–1874.

Drobek, A., Moudra, A., Mueller, D., Huranova, M., Horkova, V., Pribikova, M., Ivanek, R., Oberle, S., Zehn, D., McCoy, K.D., et al. (2018). Strong homeostatic TCR signals induce formation of self-tolerant virtual memory CD8 T cells. EMBO J. *37*, e98518.

Ebert, P.J.R., Jiang, S., Xie, J., Li, Q.J., and Davis, M.M. (2009). An endogenous positively selecting peptide enhances mature T cell responses and becomes an autoantigen in the absence of microRNA miR-181a. Nat. Immunol. *10*, 1162–1169.

Enouz, S., Carrié, L., Merkler, D., Bevan, M.J., and Zehn, D. (2012). Autoreactive T cells bypass negative selection and respond to self-antigen stimulation during infection. J. Exp. Med. *209*, 1769–1779.

Erman, B., Alag, A.S., Dahle, O., van Laethem, F., Sarafova, S.D., Guinter, T.I., Sharrow, S.O., Grinberg, A., Love, P.E., and Singer, A. (2006). Coreceptor signal strength regulates positive selection but does not determine CD4/CD8 lineage choice in a physiologic in vivo model. J. Immunol. *177*, 6613–6625.

Fontenot, J.D., Dooley, J.L., Farr, A.G., and Rudensky, A.Y. (2005). Developmental regulation of Foxp3 expression during ontogeny. J. Exp. Med. *202*, 901–906.

Fu, G., Vallée, S., Rybakin, V., McGuire, M.V., Ampudia, J., Brockmeyer, C., Salek, M., Fallen, P.R., Hoerter, J.A.H., Munshi, A., et al. (2009). Themis controls thymocyte selection through regulation of T cell antigen receptor-mediated signaling. Nat. Immunol. *10*, 848–856.

Fulton, R.B., Hamilton, S.E., Xing, Y., Best, J.A., Goldrath, A.W., Hogquist, K.A., and Jameson, S.C. (2015). The TCR's sensitivity to self peptide-MHC dictates the ability of naive CD8(+) T cells to respond to foreign antigens. Nat. Immunol. *16*, 107–117.

Gaud, G., Lesourne, R., and Love, P.E. (2018). Regulatory mechanisms in T cell receptor signalling. Nat. Rev. Immunol. *18*, 485–497.

Hogquist, K.A., and Jameson, S.C. (2014). The self-obsession of T cells: how TCR signaling thresholds affect fate 'decisions' and effector function. Nat. Immunol. *15*, 815–823.

Hogquist, K.A., Jameson, S.C., Heath, W.R., Howard, J.L., Bevan, M.J., and Carbone, F.R. (1994). T cell receptor antagonist peptides induce positive selection. Cell *76*, 17–27.

Huseby, E.S., White, J., Crawford, F., Vass, T., Becker, D., Pinilla, C., Marrack, P., and Kappler, J.W. (2005). How the T cell repertoire becomes peptide and MHC specific. Cell *122*, 247–260.

Huseby, E.S., Crawford, F., White, J., Marrack, P., and Kappler, J.W. (2006). Interface-disrupting amino acids establish specificity between T cell receptors and complexes of major histocompatibility complex and peptide. Nat. Immunol. *7*, 1191–1199.

Johnson, A.L., Aravind, L., Shulzhenko, N., Morgun, A., Choi, S.Y., Crockford, T.L., Lambe, T., Domaschenz, H., Kucharska, E.M., Zheng, L., et al. (2009). Themis is a member of a new metazoan gene family and is required for the completion of thymocyte positive selection. Nat. Immunol. *10*, 831–839.

Kasparek, P., Krausova, M., Haneckova, R., Kriz, V., Zbodakova, O., Korinek, V., and Sedlacek, R. (2014). Efficient gene targeting of the Rosa26 locus in mouse zygotes using TALE nucleases. FEBS Lett. *588*, 3982–3988.

Keck, S., Schmaler, M., Ganter, S., Wyss, L., Oberle, S., Huseby, E.S., Zehn, D., and King, C.G. (2014). Antigen affinity and antigen dose exert distinct influences on CD4 T-cell differentiation. Proc. Natl. Acad. Sci. USA *111*, 14852–14857.

Kim, P.W., Sun, Z.Y., Blacklow, S.C., Wagner, G., and Eck, M.J. (2003). A zinc clasp structure tethers Lck to T cell coreceptors CD4 and CD8. Science *301*, 1725–1728.

Kim, J.M., Rasmussen, J.P., and Rudensky, A.Y. (2007). Regulatory T cells prevent catastrophic autoimmunity throughout the lifespan of mice. Nat. Immunol. *8*, 191–197.

King, C.G., Koehli, S., Hausmann, B., Schmaler, M., Zehn, D., and Palmer, E. (2012). T cell affinity regulates asymmetric division, effector cell differentiation, and tissue pathology. Immunity *37*, 709–720.

Koehli, S., Naeher, D., Galati-Fournier, V., Zehn, D., and Palmer, E. (2014). Optimal T-cell receptor affinity for inducing autoimmunity. Proc. Natl. Acad. Sci. USA *111*, 17248–17253.

Li, Q.J., Chau, J., Ebert, P.J.R., Sylvester, G., Min, H., Liu, G., Braich, R., Manoharan, M., Soutschek, J., Skare, P., et al. (2007). miR-181a is an intrinsic modulator of T cell sensitivity and selection. Cell *129*, 147–161.

Liang, J., Lyu, J., Zhao, M., Li, D., Zheng, M., Fang, Y., Zhao, F., Lou, J., Guo, C., Wang, L., et al. (2017). Tespa1 regulates T cell receptor-induced calcium signals by recruiting inositol 1,4,5-trisphosphate receptors. Nat. Commun. *8*, 15732.

Lin, W., Truong, N., Grossman, W.J., Haribhai, D., Williams, C.B., Wang, J., Martín, M.G., and Chatila, T.A. (2005). Allergic dysregulation and hyperimmunoglobulinemia E in Foxp3 mutant mice. J. Allergy Clin. Immunol. *116*, 1106–1115.

Lo, W.L., Donermeyer, D.L., and Allen, P.M. (2012). A voltage-gated sodium channel is essential for the positive selection of CD4(+) T cells. Nat. Immunol. *13*, 880–887.

Lucas, B., Stefanová, I., Yasutomo, K., Dautigny, N., and Germain, R.N. (1999). Divergent changes in the sensitivity of maturing T cells to structurally related ligands underlies formation of a useful T cell repertoire. Immunity *10*, 367–376.

Mandl, J.N., Monteiro, J.P., Vrisekoop, N., and Germain, R.N. (2013). T cell-positive selection uses self-ligand binding strength to optimize repertoire recognition of foreign antigens. Immunity *38*, 263–274.

McKeithan, T.W. (1995). Kinetic proofreading in T-cell receptor signal transduction. Proc. Natl. Acad. Sci. USA *92*, 5042–5046.

Merry, A.H., Gilbert, R.J., Shore, D.A., Royle, L., Miroshnychenko, O., Vuong, M., Wormald, M.R., Harvey, D.J., Dwek, R.A., Classon, B.J., et al. (2003). O-glycan sialylation and the structure of the stalk-like region of the T cell co-receptor CD8. J. Biol. Chem. *278*, 27119–27128.

Molina, T.J., Kishihara, K., Siderovski, D.P., van Ewijk, W., Narendran, A., Timms, E., Wakeham, A., Paige, C.J., Hartmann, K.U., Veillette, A., et al. (1992). Profound block in thymocyte development in mice lacking p56lck. Nature *357*, 161–164.

Moody, A.M., Chui, D., Reche, P.A., Priatel, J.J., Marth, J.D., and Reinherz, E.L. (2001). Developmentally regulated glycosylation of the CD8alphabeta coreceptor stalk modulates ligand binding. Cell *107*, 501–512.

Moran, A.E., Holzapfel, K.L., Xing, Y., Cunningham, N.R., Maltzman, J.S., Punt, J., and Hogquist, K.A. (2011). T cell receptor signal strength in Treg and iNKT cell development demonstrated by a novel fluorescent reporter mouse. J. Exp. Med. *208*, 1279–1289.

Naeher, D., Daniels, M.A., Hausmann, B., Guillaume, P., Luescher, I., and Palmer, E. (2007). A constant affinity threshold for T cell tolerance. J. Exp. Med. *204*, 2553–2559.

Persaud, S.P., Parker, C.R., Lo, W.L., Weber, K.S., and Allen, P.M. (2014). Intrinsic CD4+ T cell sensitivity and response to a pathogen are set and sustained by avidity for thymic and peripheral complexes of self peptide and MHC. Nat. Immunol. *15*, 266–274.

Rudd, C.E., Trevillyan, J.M., Dasgupta, J.D., Wong, L.L., and Schlossman, S.F. (2010). The CD4 receptor is complexed in detergent lysates to a protein-tyrosine kinase (pp58) from human T lymphocytes. J. Immunol. *185*, 2645–2649.

Ruedl, C., Khameneh, H.J., and Karjalainen, K. (2008). Manipulation of immune system via immortal bone marrow stem cells. Int. Immunol. *20*, 1211–1218.

Schrum, A.G., Gil, D., Dopfer, E.P., Wiest, D.L., Turka, L.A., Schamel, W.W., and Palmer, E. (2007). High-sensitivity detection and quantitative analysis of native protein-protein interactions and multiprotein complexes by flow cytometry. Sci. STKE *2007*, pl2.

Shen, F.W., Saga, Y., Litman, G., Freeman, G., Tung, J.S., Cantor, H., and Boyse, E.A. (1985). Cloning of Ly-5 cDNA. Proc. Natl. Acad. Sci. USA *82*, 7360–7363.

Shinkai, Y., Rathbun, G., Lam, K.P., Oltz, E.M., Stewart, V., Mendelsohn, M., Charron, J., Datta, M., Young, F., Stall, A.M., et al. (1992). RAG-2-deficient mice lack mature lymphocytes owing to inability to initiate V(D)J rearrangement. Cell *68*, 855–867.

Sommers, C.L., Dejarnette, J.B., Huang, K., Lee, J., El-Khoury, D., Shores, E.W., and Love, P.E. (2000). Function of CD3 epsilon-mediated signals in T cell development. J. Exp. Med. *192*, 913–919.

Starr, T.K., Daniels, M.A., Lucido, M.M., Jameson, S.C., and Hogquist, K.A. (2003). Thymocyte sensitivity and supramolecular activation cluster formation are developmentally regulated: a partial role for sialylation. J. Immunol. *171*, 4512–4520.

Stefanová, I., Dorfman, J.R., and Germain, R.N. (2002). Self-recognition promotes the foreign antigen sensitivity of naive T lymphocytes. Nature *420*, 429–434.

Stepanek, O., Kalina, T., Draber, P., Skopcova, T., Svojgr, K., Angelisova, P., Horejsi, V., Weiss, A., and Brdicka, T. (2011). Regulation of Src family kinases involved in T cell receptor signaling by protein-tyrosine phosphatase CD148. J. Biol. Chem. *286*, 22101–22112.

Stepanek, O., Prabhakar, A.S., Osswald, C., King, C.G., Bulek, A., Naeher, D., Beaufils-Hugot, M., Abanto, M.L., Galati, V., Hausmann, B., et al. (2014). Coreceptor scanning by the T cell receptor provides a mechanism for T cell tolerance. Cell *159*, 333–345.

Swee, L.K., Tan, Z.W., Sanecka, A., Yoshida, N., Patel, H., Grotenbreg, G., Frickel, E.M., and Ploegh, H.L. (2016). Peripheral self-reactivity regulates antigen-specific CD8 T-cell responses and cell division under physiological conditions. Open Biol. *6*, 160293.

Van Laethem, F., Sarafova, S.D., Park, J.H., Tai, X., Pobezinsky, L., Guinter, T.I., Adoro, S., Adams, A., Sharrow, S.O., Feigenbaum, L., and Singer, A. (2007). Deletion of CD4 and CD8 coreceptors permits generation of alphabetaT cells that recognize antigens independently of the MHC. Immunity *27*, 735–750.

van Oers, N.S., Killeen, N., and Weiss, A. (1996). Lck regulates the tyrosine phosphorylation of the T cell receptor subunits and ZAP-70 in murine thymocytes. J. Exp. Med. *183*, 1053–1062.

Veillette, A., Bookman, M.A., Horak, E.M., and Bolen, J.B. (1988). The CD4 and CD8 T cell surface antigens are associated with the internal membrane tyrosine-protein kinase p56lck. Cell *55*, 301–308.

Wang, D., Zheng, M., Lei, L., Ji, J., Yao, Y., Qiu, Y., Ma, L., Lou, J., Ouyang, C., Zhang, X., et al. (2012). Tespa1 is involved in late thymocyte development through the regulation of TCR-mediated signaling. Nat. Immunol. *13*, 560–568.

Weber, K.S., Li, Q.J., Persaud, S.P., Campbell, J.D., Davis, M.M., and Allen, P.M. (2012). Distinct CD4+ helper T cells involved in primary and secondary responses to infection. Proc. Natl. Acad. Sci. USA *109*, 9511–9516.

Witte, T., Spoerl, R., and Chang, H.C. (1999). The CD8beta ectodomain contributes to the augmented coreceptor function of CD8alphabeta heterodimers relative to CD8alphaalpha homodimers. Cell. Immunol. *191*, 90–96.

Wyss, L., Stadinski, B.D., King, C.G., Schallenberg, S., McCarthy, N.I., Lee, J.Y., Kretschmer, K., Terracciano, L.M., Anderson, G., Surh, C.D., et al. (2016). Affinity for self antigen selects Treg cells with distinct functional properties. Nat. Immunol. *17*, 1093–1101.

Zamoyska, R., and Parnes, J.R. (1988). A CD8 polypeptide that is lost after passing the Golgi but before reaching the cell surface: a novel sorting mechanism. EMBO J. *7*, 2359–2367.

Zamoyska, R., Derham, P., Gorman, S.D., von Hoegen, P., Bolen, J.B., Veillette, A., and Parnes, J.R. (1989). Inability of CD8 alpha' polypeptides to associate with p56lck correlates with impaired function in vitro and lack of expression in vivo. Nature *342*, 278–281.

Zehn, D., Lee, S.Y., and Bevan, M.J. (2009). Complete but curtailed T-cell response to very low-affinity antigen. Nature *458*, 211–214.

STAR★METHODS

KEY RESOURCES TABLE

REAGENT or RESOURCE	SOURCE	IDENTIFIER
Antibodies		
Armenian Hamster monoclonal anti-CD3ε (clone 145-2C11) APC conjugated	BD Biosciences	Cat# 553066, RRID:AB_398529
Armenian Hamster monoclonal anti-CD3ε (clone 145-2C11) PE conjugated	BD Biosciences	Cat# 553063, RRID:AB_394596
Goat polyclonal anti-CD3ε	Santa Cruz Biotechnology	Cat# sc-1127, RRID:AB_631128
Rat monoclonal anti-CD4 (clone RM4-4)	BD Biosciences BioLegend	Cat#: 553053, RRID: AB_394588<br>Cat#: 116018, RRID: AB_2650936
Rat monoclonal anti-CD4 (clone RM4-4) FITC conjugated	BioLegend	Cat#: 116003, RRID: AB_313688
Rat monoclonal anti-CD4 (clone RM4-4) PE conjugated	BioLegend	Cat# 116006, RRID: AB_313691
Rat monoclonal anti-CD4 (clone RM4-5) biotin conjugated	BD Biosciences	Cat# 553649, RRID:AB_394969
Rat monoclonal anti-CD4 (clone RM4-5) PE conjugated	BD Biosciences	Cat# 553049, RRID:AB_394585
Rat monoclonal anti-CD4 (clone RM4-5) BV650 conjugated	BioLegend	Cat# 100545, RRID:AB_11126142
Rat monoclonal anti-CD4 (clone RM4-5) APC conjugated	BD Biosciences	Cat# 553051, RRID:AB_398528
Rat monoclonal anti-CD4 (clone RM4-5) AF700 conjugated	BD Biosciences	Cat# 557956, RRID:AB_396956
Rat monoclonal anti-CD4 (clone H129.19) PE conjugated	BD Biosciences	Cat# 553652, RRID:AB_394972
Rat monoclonal anti-CD4 (clone H129.19) FITC conjugated	BD Biosciences	Cat# 553651, RRID:AB_394971
Rat monoclonal anti-CD4 (clone GK1.5) Alexa Fluor 488 cojungated	Biolegend	Cat# 100423, RRID:AB_389302
Rabbit monoclonal anti-CD4 (clone D7D2Z)	Cell Signaling Technology	Cat# 25229, RRID:AB_2798898
Rat monoclonal anti-CD4 (clone YTS 177.9) biotin conjugated	Tomas Brdicka's lab	N/A
Rat monoclonal anti-CD8a (clone 53-5.7) FITC conjugated	BD Biosciences BioLegend	Cat# 553032, RRID:AB_394570<br>Cat# 100706, RRID:AB_312745
Rat monoclonal anti-CD8a (clone 53-5.7) BV421 conjugated	BioLegend	Cat# 100737, RRID:AB_10897101
Rat monoclonal anti-CD8a (clone 53-5.7) PE conjugated	BD Biosciences	Cat# 553033, RRID:AB_394571
Rabbit monoclonal anti-CD8a (clone D4W2Z)	Cell Signaling Technology	Cat# 98941, RRID:AB_2756376
Rat monoclonal anti-CD8b.2 (clone 53-5.8)	BD Biosciences	Cat# 553038, RRID:AB_394574
Rat monoclonal anti-CD8b.2 (clone 53-5.8) Biotin cojugated	BD Biosciences	Cat# 553039, RRID:AB_394575
Rat monoclonal anti-CD8b.2 (clone 53-5.8) FITC conjugated	BD Biosciences	Cat# 553040, RRID:AB_394576
Rat monoclonal anti-CD8b.2 (clone 53-5.8) PerCP-Cy5.5 conjugated	BioLegend	Cat# 140417, RRID:AB_2800650
Rat monoclonal anti-CD8b (clone eBioH35-17.2) PE-Cy7 conjugated	Thermo Fisher Scientific	Cat# 25-0083-82, RRID:AB_11218494

(*Continued on next page*)

Continued

REAGENT or RESOURCE	SOURCE	IDENTIFIER
Rat monoclonal anti-CD11b (clone YBM 15.1.6) biotin conjugated	Tomas Brdicka's lab	N/A
Rat monoclonal anti-CD25 (clone PC61) PE-Cy7 conjugated	BioLegend	Cat# 102016, RRID:AB_312865
Rat monoclonal anti-CD44 (clone IM7) PerCP-Cy5.5 conjugated	BioLegend	Cat# 103032, RRID:AB_2076204
Rat monoclonal anti-CD44 (clone IM7) BV650 conjugated	BioLegend	Cat# 103049, RRID:AB_2562600
Rat monoclonal anti-CD44 (clone IM7) biotin conjugated	BioLegend	Cat# 103003, RRID:AB_312954
Mouse monoclonal anti-CD45.2 (clone 104) APC-Cy7 conjugated	BD Biosciences	Cat# 560694, RRID:AB_1727492
Mouse monoclonal anti-CD45.2 (clone 104) Alexa Fluor 700 conjugated	Biolegend	Cat# 109822, RRID:AB_493731
Rat monoclonal anti-CD45R/B220 (clone RA3-6B2) biotin conjugated	BD Biosciences	Cat# 553085, RRID:AB_394615
Rat monoclonal anti-CD62L (clone MEL-14) PE-Cy7 conjugated	BioLegend	Cat# 104418, RRID:AB_313103
Armenian Hamster monoclonal anti-CD69 FITC conjugated	BD Biosciences	Cat# 553236, RRID:AB_394725
Mouse monoclonal anti-Lck (clone 3a5)	Santa Cruz Biotechnology	Cat# sc-433, RRID:AB_627880
Mouse monoclonal anti-Lck (clone 3a5) PE conjugated	Santa Cruz Biotechnology	Cat# sc-433 PE, RRID: N/A
Mouse monoclonal anti-MHCI (H2Kb; clone Y3.8)	Ed Palmer's lab, University of Basel	N/A
Armenian Hamster monoclonal anti-TCRβ (clone H57-597) APC conjugated	BD Biosciences Biolegend	Cat# 553174, RRID:AB_398534<br>Cat# 109212, RRID:AB_313435
Rabbit monoclonal anti-Phospho-p44/42 MAPK (Erk1/2) (Thr202/Tyr204) (clone D13.14.4E)	Cell Signaling Technology	Cat# 4370, RRID:AB_2315112
Mouse monoclonal anti-Src, non-phospho (Tyr416) (clone 7G9)	Cell Signaling Technology	Cat# 2102, RRID:AB_331358
Rabbit polyclonal anti-Phospho-Src Family (Tyr416)	Cell Signaling Technology	Cat# 2101, RRID:AB_331697
Mouse monoclonal anti-Phosphotyrosine (clone 4G10) PE conjugated	Millipore	Cat# FCMAB323PE, RRID:AB_10805942
Mouse monoclonal anti-CD247 phospho (Tyr142) (clone K25-407.69) PE conjugated	BD Biosciences	Cat# 558448, RRID:AB_647237
Rabbit monoclonal anti-Phospho-Zap-70 (Tyr319)/Syk (Tyr352) (clone 65E4)	Cell Signaling Technology	Cat# 2717, RRID:AB_2218658
Mouse monoclonal anti-Lamin B1 (clone 119D5-F1)	Santa Cruz Biotechnology	Cat# sc-56143, RRID:AB_2136302
Mouse monoclonal anti-Zap70 (clone 1E7.2) Alexa Fluor 488 conjugated	Thermo Fisher Scientific	Cat# MHZAP7020, RRID:AB_10375316
Rabbit polyclonal anti-GAPDH	Sigma-Aldrich	Cat# G9545, RRID:AB_796208
Donkey polyclonal anti-Rabbit IgG (H+L) Highly Cross-Adsorbed Secondary Antibody, Alexa Fluor 555 conjugated	Thermo Fisher Scientific	Cat# A-31572, RRID:AB_162543
Goat polyclonal anti-Rabbit IgG (H+L) Highly Cross-Adsorbed Secondary Antibody, Alexa Fluor 555 conjugated	Thermo Fisher Scientific	Cat# A-21429, RRID:AB_2535850
Goat polyclonal anti-Rabbit IgG (H+L) Highly Cross-Adsorbed Secondary Antibody, Alexa Fluor 488 conjugated	Thermo Fisher Scientific	Cat# A-11034, RRID:AB_2576217

(*Continued on next page*)

Continued

REAGENT or RESOURCE	SOURCE	IDENTIFIER
Goat polyclonal anti-mouse IgG (H+L) Secondary Antibody, HRP conjugated	Jackson ImmunoResearch Labs	Cat# 115-035-003, RRID:AB_10015289
Donkey polyclonal anti-Goat IgG (H+L) antibody, HRP conjugated	Jackson ImmunoResearch Labs	Cat# 705-035-003, RRID:AB_2340390
Goat polyclonal anti-rabbit IgG (H+L) Secondary Antibody, HRP conjugated	Jackson ImmunoResearch Labs	Cat# 111-035-003, RRID:AB_2313567
Bacterial and Virus Strains		
Listeria monocytogenes (transfected with empty pPL1,pPL2)	(Zehn et al., 2009)	N/A
Listeria monocytogenes expressing SIINFEKL (OVA) peptide (transfected with pPL1,pPL2 carrying OVA peptide coding sequence)	(Zehn et al., 2009)	N/A
Listeria monocytogenes expressing SIITFEKL (T4) peptide (transfected with pPL1,pPL2 carrying T4 peptide coding sequence)	(Zehn et al., 2009)	N/A
Listeria monocytogenes expressing SIIQFEHL (Q4H7) peptide (transfected with pPL1,pPL2 carrying Q4H7 peptide coding sequence)	(King et al., 2012)	N/A
Listeria monocytogenes expressing FEAQKAKANKAVD (3K) peptide (transfected with pPL1,pPL2 carrying 3K peptide coding sequence)	This paper	N/A
Listeria monocytogenes expressing FEAAKAKANKAVD (P2A) peptide (transfected with pPL1,pPL2 carrying P2A peptide coding sequence)	This paper	N/A
Listeria monocytogenes expressing FAAQKAKANKAVD (P-1A) peptide (transfected with pPL1,pPL2 carrying P-1A peptide coding sequence)	This paper	N/A
Chemicals, Peptides, and Recombinant Proteins		
AccuCheck Counting Beads	Thermo Fisher Scientific	Cat# PCB100
Qdot 605 Streptavidin Conjugate	Invitrogen	Cat# Q10103MP
CML Latex Beads, 4% w/v, 5 μm	Thermo Fisher Scientific	Cat# C37255
Nonidet P 40 Substitute	Sigma Aldrich	Cat# 74385
n-Dodecyl-beta-Maltoside Detergent	Thermo Fisher Scientific	Cat# 89903
cOmplete, EDTA-free Protease Inhibitor Cocktail Tablets	Roche	Cat# 05056489001
4-(2-Aminoethyl)benzenesulfonyl fluoride hydrochloride	Sigma Aldrich	Cat# A8456
PhosSTOP	Roche Molecular Systems, Inc	Cat# 04906837001
Amersham Protran 0.45 NC nitrocellulose western blotting membranes	GE Healthcare	Cat# 10600002
Streptavidin Mag Sepharose	GE Healthcare	Cat# 28985738
LPS *E.Coli* O111:B4	Sigma Aldrich	Cat# LPS25
OVA peptide (SIINFEKL)	Eurogentec	Ref# AS-60193-1
T4 peptide (SIITFEKL)	Eurogentec	Ref# AS-64403
Q4H7 peptide (SIIQFEHL)	Eurogentec	Ref# AS-64405
3K peptide (FEAQKAKANKAVD)	Peptides and Elephants	N/A
P2A peptide (FEAAKAKANKAVD)	Peptides and Elephants	N/A

(*Continued on next page*)

Continued

REAGENT or RESOURCE	SOURCE	IDENTIFIER
P-1A peptide (FAAQKAKANKAVD)	Peptides and Elephants	N/A
(+)-Biotin N-hydroxysuccinimide ester	Sigma Aldrich	Cat# H1759
Sephadex® G-25	Sigma Aldrich	Cat# S5772
Critical Commercial Assays		
LIVE/DEAD Fixable Near-IR Dead Cell Stain Kit	Thermo Fisher Scientific	L34976
CellTrace CFSE Cell Proliferation Kit	Thermo Fisher Scientific	C34554
Untouched Mouse CD8 Cells Kit	Dynabeads	Cat# 11417D
Untouched Mouse CD4 Cells Kit	Dynabeads	Cat# 11415D
Biotin Binder	Dynabeads	Cat# 11047
EasySep Mouse CD8 T Cell Enrichment Kit	Stem Cell	Cat# 19753A
RNA Clean and Concentrator^{-5}	Zymo Research	Cat# R1013
Neuraminidase from Vibrio cholera, type II	Sigma Aldrich	Cat# N6514
Experimental Models: Cell Lines		
Lutz	N/A	N/A
Experimental Models: Organisms/Strains		
Mouse: C57BL/6J	Animal Facility of Institute of Molecular Genetics	JAX 000664
Mouse: C57BL/6J CD45.1	(Shen et al., 1985)	JAX 002014
Mouse: CD3ε$^{-/-}$	(Sommers et al., 2000)	JAX 004177
Mouse: CD8.4	(Erman et al., 2006)	N/A
Mouse: OT-I Rag2$^{-/-}$	(Hogquist et al., 1994; Shinkai et al.1992)	N/A
Mouse: B3K508 Rag2$^{-/-}$	(Huseby et al., 2005; Shinkai et al., 1992)	N/A
Mouse: FoxP3$^{-/-}$	(Lin et al., 2005)	JAX 019933
Mouse: Nur77-GFP	(Moran et al., 2011)	JAX 016617
Mouse: FoxP3-GFP	(Fontenot et al., 2005)	N/A
Mouse: FoxP3-DTR	(Kim et al., 2007)	JAX 016958
Mouse: Lck$^{-/-}$	This paper	N/A
Oligonucleotides		
CD8 Forward – CCGTGGCTCAGTGAAGGGG	Sigma Aldrich	N/A
CD8' Reverse – CTGACTAGCGGCTGTGGTAGC	Sigma Aldrich	N/A
CD8 Full length Reverse – CATTTGCAAACACGCTTTCGGCTC	Sigma Aldrich	N/A
CD8 Total Reverse - CTTGCCTTCCTGTCTGACTAGC	Sigma Aldrich	N/A
gRNA for Lck$^{-/-}$ generation: TTGCTGTCCAGTGGGACTAT **GGG**	N/A	N/A
Software and Algorithms		
Source code for 'Lck come&stay/signal duration' model (MATLAB)	This paper (Data S1)	N/A
GraphPad Prism 5.04	GraphPad Software	N/A
FlowJo V9 and V10	FlowJo, LCC	N/A
R Studio V1.2.1335	RStudio, Inc.	N/A
Tescan Q-Phase software V7.727	Tescan Orsay Holding, a.s	N/A
Fiji (ImageJ version 1.52i)	Open Source	N/A
MATLAB	MathWorks	N/A

(*Continued on next page*)

Continued

REAGENT or RESOURCE	SOURCE	IDENTIFIER
Other		
LSRII	BD Biosciences	N/A
CantoII	BD Biosciences	N/A
FACSymphony	BD Biosciences	N/A
LSRFortessa	BD Biosciences	N/A
Influx Sorter	BD Biosciences	N/A
Cytek™ Aurora	Cytek	N/A
LI-COR Odyssey infrared imaging system	LI-COR Biosciences	N/A
Azure c200 imaging system	Azure Biosystems	N/A
Z2 Coulter Counter Analyzer	Beckman Coulter	N/A
LightCycler® 480 Instrument II	Roche Molecular Systems, Inc	N/A

LEAD CONTACT AND MATERIALS AVAILABILITY

Further information and requests for resources and reagents should be directed to and will be fulfilled by the Lead Contact, Ondrej Stepanek (ondrej.stepanek@img.cas.cz). All unique/stable reagents (i.e., *Listeria monocytogenes* strains and *Lck*$^{-/-}$ mouse strain) generated in this study are available from the Lead Contact with a completed Materials Transfer Agreement.

EXPERIMENTAL MODEL AND SUBJECT DETAILS

Mice

All mice had C57BL/6J background and were 6-20 weeks old. Both males and females were used for experiments. For adoptive T cell transfers, only females were used as donors. *Foxp3*-deficient mice and littermate controls were analyzed at the age of 2-3 weeks old. Mice were bred in our SPF facilities (University Hospital Basel and Institute of Molecular Genetics) in accordance with Cantonal and Federal laws of Switzerland and the laws of the Czech Republic. Animal protocols were approved by the Cantonal Veterinary Office of Basel-Stadt, Switzerland, and the Czech Academy of Sciences, Czech Republic. The used strains were: Ly5.2, Ly5.1 (Shen et al., 1985), *Cd3ε*$^{-/-}$ (Sommers et al., 2000), CD8.4 (Erman et al., 2006), OT-I *Rag2*$^{-/-}$ (Hogquist et al., 1994; Shinkai et al., 1992), B3K508 *Rag2*$^{-/-}$ (Huseby et al., 2005; Shinkai et al., 1992), *Foxp3*-deficient (Lin et al., 2005), *Nur77*-GFP (Moran et al., 2011), *Foxp3*-GFP (Fontenot et al., 2005), *Foxp3*-DTR (Kim et al., 2007). Strains CD8.4 and *Nur77*-GFP were bred to obtain strain CD8.4 *Nur77*-GFP. Mice were kept in the animal facility with 12 hours of light and dark cycle with food and water *ad libitum*.

Lck$^{-/-}$ mice were generated on C57BL/6J background in the Czech Centre for Phenogenomics, Institute of Molecular Genetics, ASCR. The mice were generated by pronuclear microinjection of Cas9 mRNA and gRNA (TTGCTGTCCAGTGGGACTAT GGG) at concentration 100 ng/μl, into one-cell-stage murine embryos as described previously (Kasparek et al., 2014). The *Lck*$^{+/-}$ mice were backcrossed on C57BL/6J background at least 5 generations. The mice bear a deletion of 75-82 nt in the exon 2 (transcript ID: ENSMUST00000067240.10) of *Lck* gene, resulting in a frameshift and early termination of translation.

Bone marrow-derived dendritic cells (BMDC)

Dendritic cells were derived from fresh or immortalized (Drobek et al., 2018; Ruedl et al., 2008) hematopoietic stem cells. The cells were seeded on 100 mm non-treated plates and maintained in D-MEM (Sigma Aldrich) containing 10% FBS (GIBCO), 100 U/ml penicillin (BB Pharma), 100 μg/ml streptomycin (Sigma Aldrich), 40 μg/ml gentamicin (Sandoz) and 2% of supernatant from Lutz cells for 7 days at 5% CO_2, 37°C. The cells were split and media was refreshed every 2-3 days. On day 7, 100 ng/ml LPS (Sigma Aldrich) was added and the cells were seeded on 96-well plate at frequency 0.5×10^6 cells/ml.

METHOD DETAILS

Flow cytometric immunoprecipitation assay

Peripheral CD8$^+$ and CD4$^+$ T cells were enriched by magnetic beads (Dynabeads) and FACS-sorted CD4$^+$CD8$^+$ CD3^{LOW} preselection thymocytes were used for the flow cytometric immunoprecipitation assay. 10^7 cells were lysed in 50 μl lysis buffer (1% NP-40S, 50 mM Tris pH 7.4, 150 mM NaCl, AEBSF protease inhibitor (Sigma Aldrich)) for 30 min on ice. 75,000 CML beads (Invitrogen) coupled to anti-CD4 (RM4.4), anti-CD8β (53-5.8), or anti-MHCI (Y3.8) antibodies, as described previously (Schrum et al., 2007), were added to the lysate and incubated for 1 hr at 4°C. Beads were washed 3x in the lysis buffer and stained with different PE-conjugated antibodies specific to CD4 (H129.19, 8 μg/mL), CD8α (53-6.7, 20 μg/mL), or LCK (3A5, 67 μg/mL) at saturating concentrations (30 min, on ice)

and analyzed by flow cytometry. The geometric mean fluorescence intensities (gMFI) were taken as the measure of the antibody binding. The CD8, CD8.4 or CD4-LCK coupling ratio was calculated as LCK signal to CD8 or CD4 signal (after subtracting respective background signal measured from control anti-MHCI beads) and adjusted for the PE/antibody ratio.

Analysis of soluble and insoluble fractions

10^7 thymocytes or peripheral LN cells were lysed in 50 μl lysis buffer (1% NP-40S, 50 mM Tris pH 7.4, 150 mM NaCl, complete protease inhibitor cocktail (Roche)) for 30 min on ice. The samples were centrifuged and the soluble fraction was separated from the insoluble fraction. The insoluble fraction was washed in the lysis buffer and then resuspended in the same volume of the lysis buffer as the separated supernatant. Concentrated Laemmli sample buffer was added to the final concentration: 62.5 mM Tris, 10% glycerol, 1% SDS, 0.005% Bromphenol Blue, 50 mM DTT. The samples were incubated at 94°C (3 min). The NP-40S-insoluble fraction was dissolved by sonication. The samples were immunoblotted using antibodies to CD4 (D7D2Z), CD8α (D4W2Z), LCK (3a5), CD3ε (goat polyclonal), GAPDH (rabbit polyclonal), lamin B1 (119D5-F1) and visualized with HRP conjugated goat anti-mouse, goat anti-rabbit or donkey anti-goat antibodies using an Azure c200 imaging system.

Immunoprecipitation of surface coreceptors

Total thymocytes or peripheral $CD8^+$ and $CD4^+$ T cells enriched by negative magnetic beads separation (Dynabeads, StemCell) were used for immunoprecipitation. 2-3 × 10^7 of live cells were stained with biotinylated anti-CD8β (53-5.8) or anti-CD4 (H129.19) antibodies. Cells were lysed in 1 mL lysis buffer (1% Lauryl-β-D-maltoside (Thermo Fisher Scientific), 30 mM Tris, 120 mM NaCl, 2 mM KCl, 10% glycerol, 50x complete protease inhibitors (Roche)), lysate was cleared by centrifugation and supernatant was incubated with Streptavidin Mag Sepharose (GE Healthcare) for 2 hr at 4°C. Washed beads were lysed in Laemmli sample buffer. Samples were subjected to immunoblotting with rabbit mAb CD8α (D4W2Z) or CD4 (D7D2Z) and LCK (3A5) and visualized with goat anti-rabbit or goat anti-mouse antibodies conjugated with AF680 on LI-COR Odyssey infrared imaging system. Quantification analysis was done using FIJI software.

Neuraminidase treatment

Total 10^7 thymocytes or peripheral T cells were treated by 0.08 U of type II neuraminidase from *Vibrio cholerae* (Sigma) for 1 hour at 37°C in RPMI (Sigma). The cells were then either stained for flow cytometry analysis: using saturating concentration of anti-CD8β-APC (4 μg/ml, clone 53-5.8), together with anti-CD8α-AlexaFluor488 (clone 53-6.7) at a saturating concentration of 20 μg/ml or a non-saturating concentration of 32 ng/ml; or lysed in 300 μL lysis buffer (see immunoprecipitation of cell surface coreceptors for details) for 30 minutes on ice and centrifuged (20,000 g, 5 min). The supernatant was diluted in 4x concentrated Laemmli sample buffer. Samples were subjected to immunoblotting with rabbit mAb CD8α (D4W2Z) and visualized with goat anti-rabbit antibody conjugated with AF680 on LI-COR Odyssey infrared imaging system. Quantification analysis was done using FIJI software.

Quatitative PCR

RNA was isolated from total thymocytes or sorted $CD4^+$ or $CD8^+$ peripheral T cells using TRIzol™ LS (Ambien) and RNA Clean & Concentrator^{-5} (Zymo Research). The RNA was converted to cDNA using RevertAid Reverse Transcriptase (Thermo Fisher Scientific). Quantitative PCR analysis was performed using LightCycler® 480 SYBR Green I Master (Roche) in LightCycler® 480 Instrument II (Roche).

Mathematical model

The analytical solution of the 'LCK come&stay/signal duration' mathematical model was described previously (Stepanek et al., 2014). The model predicts number of occupied and triggered TCRs at any time as described previously. The parameters for the model were used as described previously for thymocytes (Stepanek et al., 2014) or obtained experimentally for LN T cells (Figures S1J–S1M; Table S1). The calculations were performed in MATLAB (MathWorks). The script is included as Data S1.

T cell enrichment

CD8, CD8.4, or CD4 T cells were enriched by negative selection using kits from Dynabeads or StemCell or Dynabeads Biotin Binder kit using biotinylated anti-CD4 (YTS 177.9), anti-CD11b (YBM 15.1.6), anti-CD45R/B220 (RA3-6B2), and eventually anti-CD44 (IM7) antibodies. The anti-CD4 and anti-CD11b antibodies were produced and biotinylated in house using (+)-Biotin *N*-hydroxysuccinimide ester (Sigma Aldrich) in bicarbonate buffer. The excess biotin was separated from the antibody using Sephadex G-25 (Sigma Aldrich).

Flow cytometry analysis and sorting

Live cells were stained with relevant antibodies and LIVE/DEAD Near-IR viability dye on ice. For intracellular staining, cells were fixed in 4% formaldehyde (15 min, RT) immediately after isolation, permeabilized by 90% methanol (30 min, on ice) and stained with indicated antibodies at RT. The cells were analyzed on LSRII, FACSCantoII, LSRFortessa, FACSymphony (BD Bioscience) or on Aurora (Cytek). The cells were sorted on Influx sorter (BD Bioscience). For the analysis of pTCRζ and pZAP70 and overall tyrosine phosphorylation in *Lck*+/+, $Lck^{+/-}$ and $Lck^{-/-}$ mice, the LN cells from these three mice were fixed and permeabilized and multiplexed by

separate staining of CD45.2 (antibody clone 104) conjugated with two different fluorophores (AF700, APC-Cy7) or their combination and mixed prior to the staining with phospho-specific antibodies. The usage of particular fluorophores for particular mouse stain was different for each experiment to avoid any possible effects of the fluorophores on the results.

Determination of LCK phosphorylation status

LCK was immunoprecipitated from untreated or PP2-treated B3K508 and OT-I T cells and analyzed by immunoblotting. Signals from antibodies recognizing phosphorylated and non-phosphorylated LCK (Y394) were normalized to total LCK and the percentage of phosphorylated molecules was calculated as previously described (Stepanek et al., 2011, 2014). Because we did not observe a difference in the pLCK/total LCK ratios between B3K508 and OT-I T cells, we pooled the data from B3K508 and OT-I T cells to estimate the percentage of phosphorylated LCK molecules in peripheral T cells.

Determination of TCR and coreceptor levels

The number of the TCR molecules and CD4 and CD8 coreceptors on the peripheral T cells was determined by flow cytometry as previously described (Stepanek et al., 2014).

Antigen presentation assay

BMDCs were cultivated from fresh or immortalized (Drobek et al., 2018; Ruedl et al., 2008) hematopoietic stem cells. The dendritic cells were pulsed with indicated concentration of indicated peptides and mixed with isolated T cells or thymocytes in ratio 1:1 or 1:2 and co-cultured in RPMI (Sigma Aldrich) containing 10% FBS (GIBCO), 100 U/ml penicillin (BB Pharma), 100 μg/ml streptomycin (Sigma Aldrich), 40 μg/ml gentamicin (Sandoz) for indicated period of time (pERK1/2 analysis) or over-night (CD25 and CD69 analysis). The EC_{50} values for the CD69 upregulation assay were calculated using non-liner fit (log(agonist) versus response–Variable slope (four parameters)) in GraphPad Prism 5. The cell numbers were counted using Z2 Coulter Counter Analyzer (Beckman Coulter).

Listeria infection

The LN T cells were isolated from B3K508 and OT-I mice and loaded with CFSE. The CFSE labeling of the cells was verified by flow cytometry and the cells were simultaneously counted using AccuCheck Counting beads (Thermo Fisher Scientific). The cells were adoptively transferred to Ly5.1 congenic host mice. The mice were injected with 5000 CFU of transgenic *Listeria monocytogenes* (Lm) expressing peptides OVA, T4, 3K and P-1A as described previously (Keck et al., 2014; Zehn et al., 2009). The expression of CD25, CFSE dilution, and cell expansion were analyzed 4 days after infection. Preceding flow cytometry analysis, the cells were enriched using negative magnetic bead separation kit for $CD4^+$ or $CD8^+$ cells (for samples with adoptively transferred B3K508 and OT-I cells respectively; Dynabeads).

Quantitative phase imaging

Cells isolated from WT and CD8.4 mice were stained with anti-CD4-Alexa Fluor 488 (RM4-5), anti-CD8α-Brilliant Violet 421 (53-5.7) and anti-CD44 (IM7, biotin conjugate) antibodies, further stained with Qdot 605 Streptavidin Conjugate and observed under Tescan Q-Phase microscope (Tescan Orsay Holding) equipped with Nikon DS-Qi1Mc camera. Samples were kept in a climatic box with stable 37°C and 5% CO2 and imaged using 20x/0.5 dry objective. Data were analyzed using Tescan Q-Phase software and RStudio.

Antibody competition binding assay

0.5×10^6 cells isolated from LN of C57BL/6J mice were stained first with saturating concentration of anti-CD4 antibody conjugated with FITC (RM4-4 or H129.19), then washed three times and stained with saturating concentrations of anti-CD4 antibody conjugated with PE (RM4-4 or H129.19). Single stained cells served as controls. The fluorescence was analyzed using a FACSymphony (BD Biosciences).

QUANTIFICATION AND STATISTICAL ANALYSIS

Data were displayed as Mean or Mean + SEM. Statistical analysis was performed with the two-tailed Mann-Whitney test, the one-value t test (after the data passed the Kolmogorov–Smirnov normality test), paired t test, or Kruskall-Wallis test whenever appropriate as indicated. Statistical analysis was performed in Graphpad Prism 5. The quantification of western blots was performed in FIJI. The dry mass analysis was performed in Tescan Q-Phase software and evaluated in RStudio. The area under curve was calculated in Excel.

DATA AND CODE AVAILABILITY

The 'LCK come&stay/signal duration' mathematical model script is included as Data S1. Software used is described in Key Resources Table.

Cell Reports, Volume *30*

Supplemental Information

Dynamics of the Coreceptor-LCK Interactions during T Cell Development Shape the Self-Reactivity of Peripheral CD4 and CD8 T Cells

Veronika Horkova, Ales Drobek, Daniel Mueller, Celine Gubser, Veronika Niederlova, Lena Wyss, Carolyn G. King, Dietmar Zehn, and Ondrej Stepanek

Figure S1

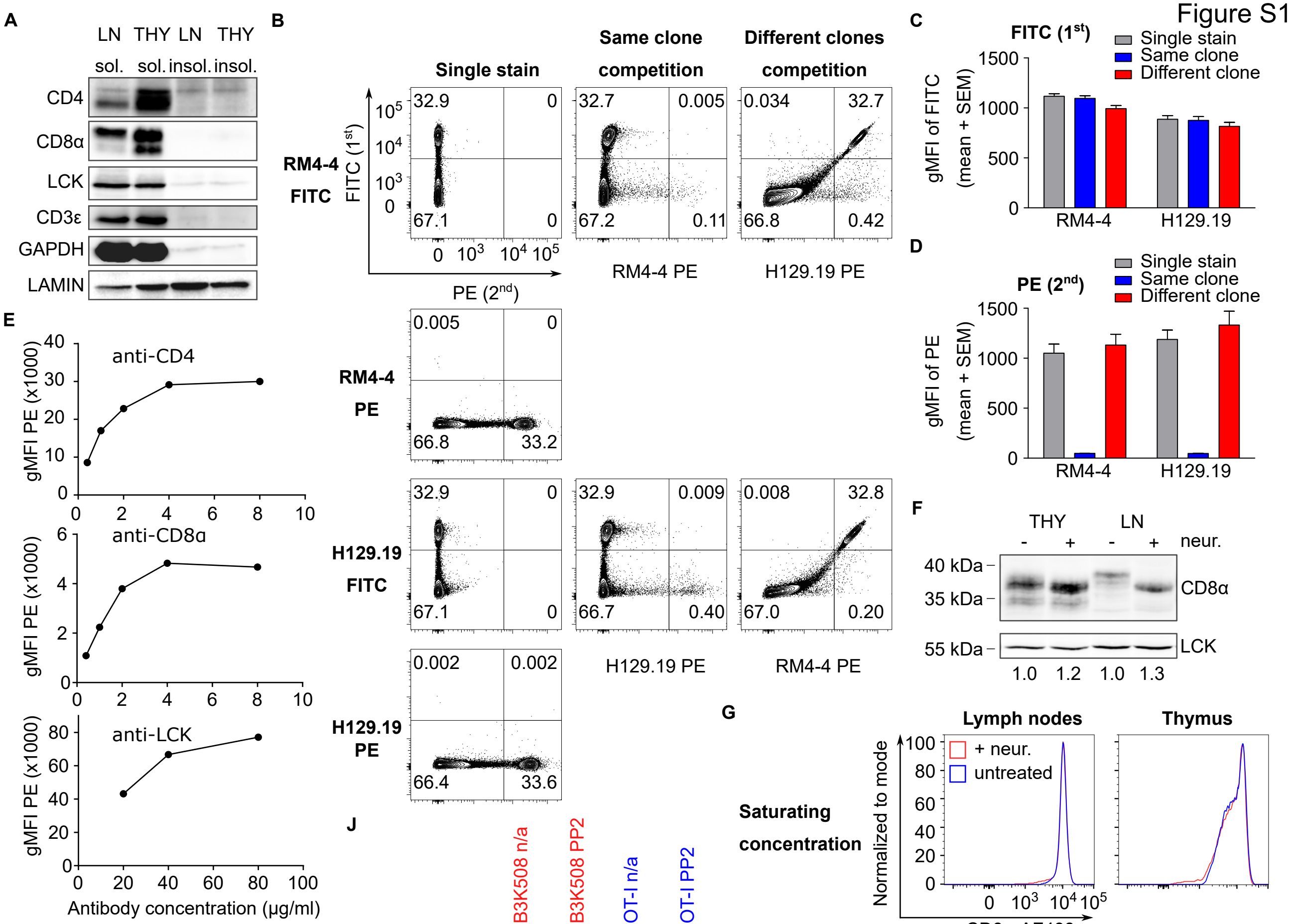

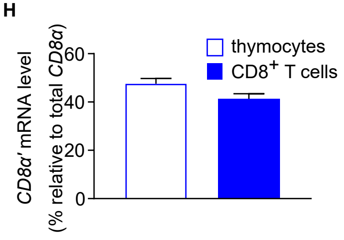

K

pLCK/total LCK	B3K508	OT-I
Exp 1	1	1.63
Exp 2	1	0.61
Exp 3	1	0.96
Exp 4	1	0.77
**Average**	1	0.99

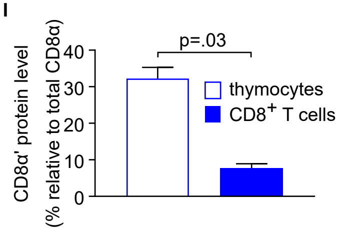

L

% pLCK	B3K508	OT-I
Exp 1	17	23
Exp 2	-22	26
Exp 3	25	6
Exp 4	-74	33
**Average**	**21.7**	
**SEM**	**3.8**	

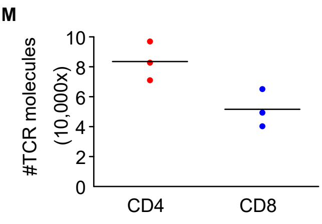

Fig. S1. Related to Figure 1.

(A) Soluble and insoluble fraction of lysate (1% NP-40S) from full LN and thymus were analyzed by immunoblotting. The membranes were stained with antibodies specific for CD4 (D7D2Z), CD8α (D4W2Z), LCK (3a5), CD3ε (goat polyclonal), GAPDH (rabbit polyclonal), LAMIN B1 (119D5-F1) (B-D) The LN T cells were stained first with FITC-conjugated antibodies (H129.19 or RM4-4). After the antibody was washed out the cells were stained with PE-conjugated antibodies specific for CD4 (H129.19 or RM4-4) in all 4 combinations. To address whether these two antibody clones compete with each other, we compared the fluorescence intensities from single stained cells, cell stained sequentially with the same antibody clone coupled to two different fluorophores, and cells stained sequentially with two different clones coupled to two different fluorophores. (B) A representative experiment out of 3 independent experiments. (C) gMFI levels of FITC. Mean + SEM, n = 3 mice (D) gMFI levels of PE. Mean + SEM, n = 3 mice. (E) Titration of antibodies used for the analysis of FC-IP experiments. A representative experiment out of 2 independent experiments is shown. (F) Thymocytes and peripheral T cells were treated with neuraminidase or not and analyzed by immunoblotting. The membranes were probed with antibodies specific for CD8α (D4W2Z) and LCK (3a5). Change in the anti-CD8α signal (normalized to LCK expression) relative to non-treated controls is indicated. Representative experiment out of 4 independent experiments. (G) Thymocytes and LN cells were treated with neuraminidase or not and analyzed by flow cytometry. The cells were stained with a saturating concentration of anti-CD8β-APC (4 µg/ml, clone 53-5.8), together with anti-CD8α-AlexaFluor488 (clone 53-6.7) at a saturating concentration of 20 µg/ml or a non-saturating concentration of 32 ng/ml. The CD8α signal on CD8β+ cells is shown. A representative experiment out of 2 in total. (H) Quantitative PCR analysis was performed on total thymocytes vs peripheral $CD8^+$ T cells. Mean + SEM, n = 3 mice (I) Quantitative analysis of surface CD8α' protein level in thymus and in peripheral $CD8^+$ T cells form experiments in Fig.1B-C. Mean + SEM, n = 5 mice in 4 independent experiments. (J-L) LCK was immunoprecipitated from lysates from non-treated (NT) or 20 µM PP2-treated B3K508 or OT-I peripheral LN T cells. Phosphorylation of LCK was analyzed by immunoblotting using simultaneous staining with antibodies specific for phosphorylated or non-phosphorylated Y394. The membrane was re-probed with antibody to total LCK. (J) Representative image out of 4 independent experiments is shown. (K) Signal of phospho-LCK to total LCK for B3K508 and OT-I T cells is shown. Data were normalized to B3K508 in each experiment. n = 4. (L) Percentage of phosphorylated LCK molecules in B3K508 and OT-I peripheral T cells was calculated for each experiment. For B3K508, two calculations led to negative values that were excluded from the final calculation, because such values make no biological and mathematical sense and were apparently caused by the error in the measurement. The average of the remaining 6 values was used for the estimation of the percentage of phosphorylated LCK molecules. (M) The peripheral $CD4^+$ and $CD8^+$ T cells were analyzed for their expression of CD3/TCR by flow cytometry.

Figure S2

A

TCR	Ligand	Selection*	Affinity (KD, µM)
OT-I	OVA	Negative	54*
OT-I	T4	Part. negative/ threshold	444*
OT-I	Q4H7	Positive	847*
B3K508	3K	Negative	29#
B3K508	P2A	Negative	175#
B3K508	P-1A	Part. negative/ threshold	263#

B

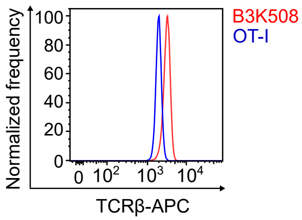

D

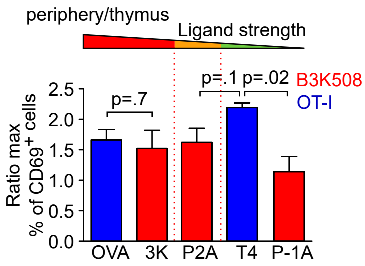

G

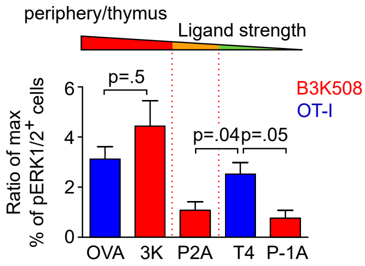

C

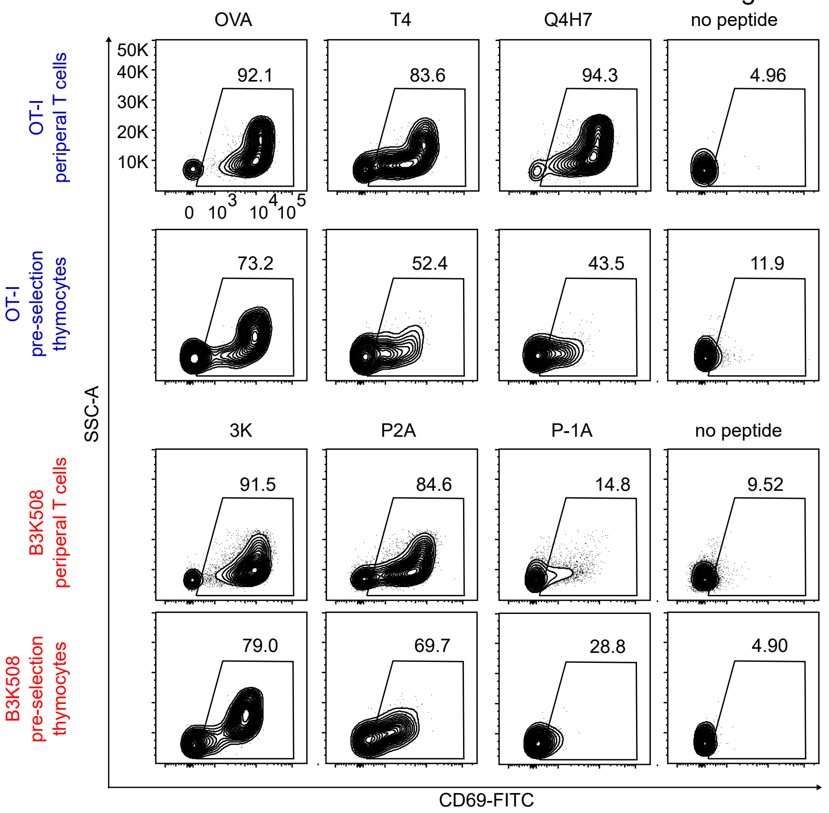

E

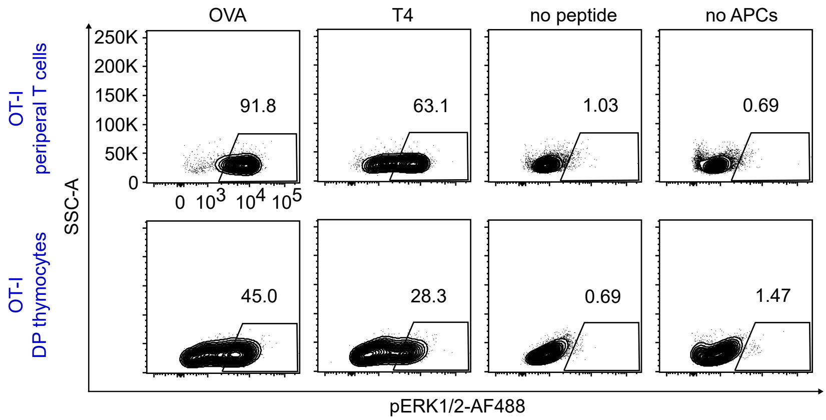

F

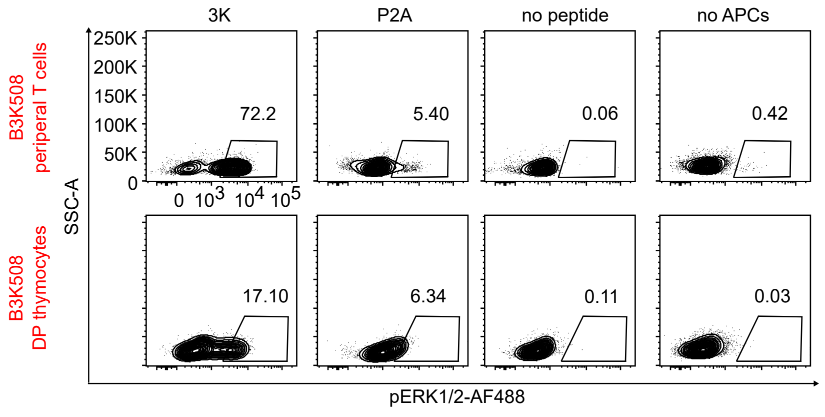

Fig. S2. Related to Figure 2.

(A) List of ligands to corresponding transgenic TCR used in Fig. 2A-D. The measured dissociation constant *(Stepanek et al., 2014) #(Huseby et al., 2006) and their ability to induce the thymic selection is denoted. (B) TCRβ expression on $CD8^+$ $CD44^-$ OT-I and $CD4^+$ $CD44^-$ B3K508 T cells. A representative experiment out of 4 in total. (C) Representative data from experiments shown in Fig. 2A are shown. (D) The ratio of maximum activation (% of $CD69^+$ cells) of the T cells in periphery vs. thymus. Mean + SEM, n = 4 mice in 4 independent experiments. (E-F) Alternative analysis of experiments shown in Figure 2. Representative data from experiments shown in Fig. 2C are shown. (G) The ratio of maximum activation (% of $CD69^+$ cells) of the T cells in periphery vs thymus. Mean + SEM. n = 6-10 mice in 6-10 independent experiments.

Figure S3

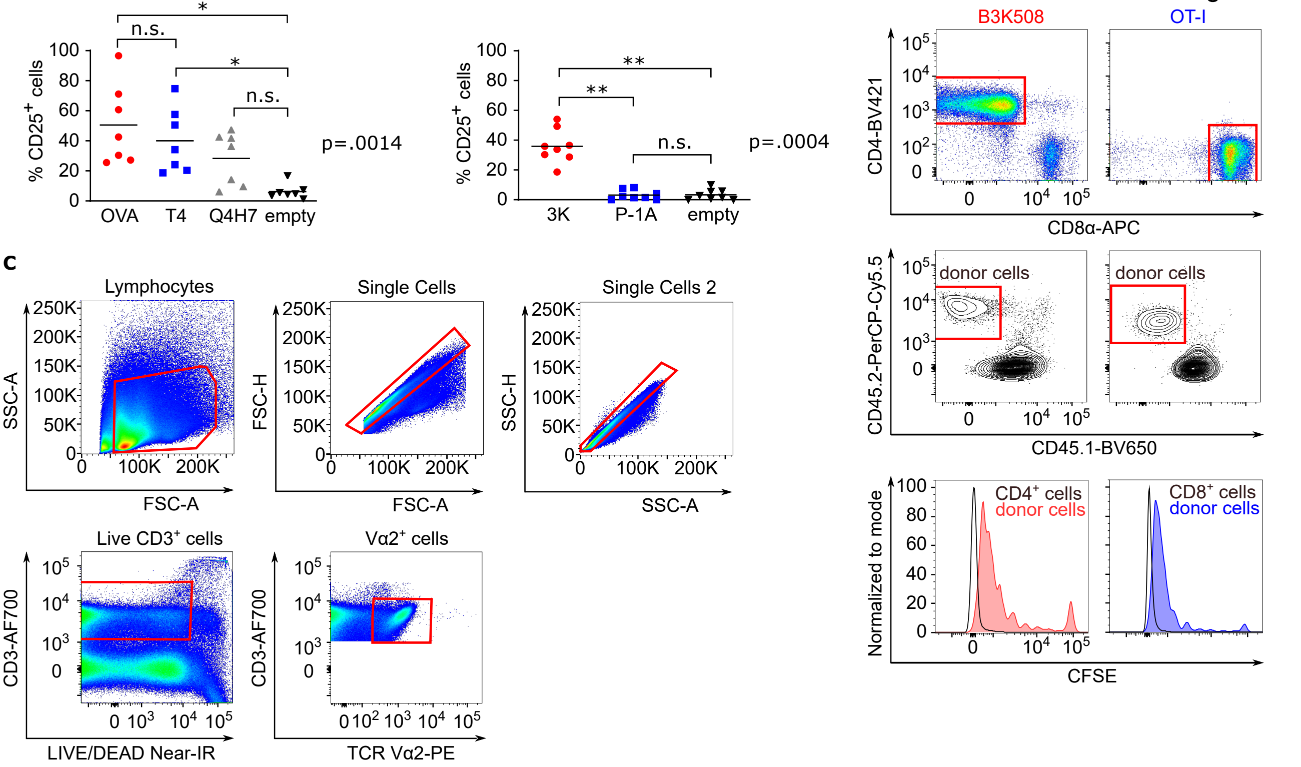
A
% CD25+ cells
OVA
T4
Q4H7
empty
n.s.
*
p=.0014
B
3K
P-1A
empty
**
n.s.
p=.0004
C
Lymphocytes
Single Cells
Single Cells 2
SSC-A
FSC-A
FSC-H
Live CD3+ cells
Vα2+ cells
CD3-AF700
LIVE/DEAD Near-IR
TCR Vα2-PE
D
B3K508
OT-I
CD4-BV421
CD8α-APC
donor cells
CD45.2-PerCP-Cy5.5
CD45.1-BV650
CD4+ cells
donor cells
CD8+ cells
donor cells
Normalized to mode
CFSE

Fig. S3. Related to Figure 3.

(A-B) Quantification of % $CD25^+$ monoclonal T cells from experiment in Fig.3A-B. Statistical analysis was performed using Kruskal-Wallis test with Dunn's Multiple Comparison post-tests (* $p<0.05$, ** $p<0.01$). (C-D) Gating strategy for the experiments in Fig.3A-B. (C) Initial gating strategy used for all samples. (D) Additional gating strategy for B3K508 (Lm-3K) or OT-I (Lm-T4) cells. The CFSE signals on donor cells are shown as histograms.

Figure S4

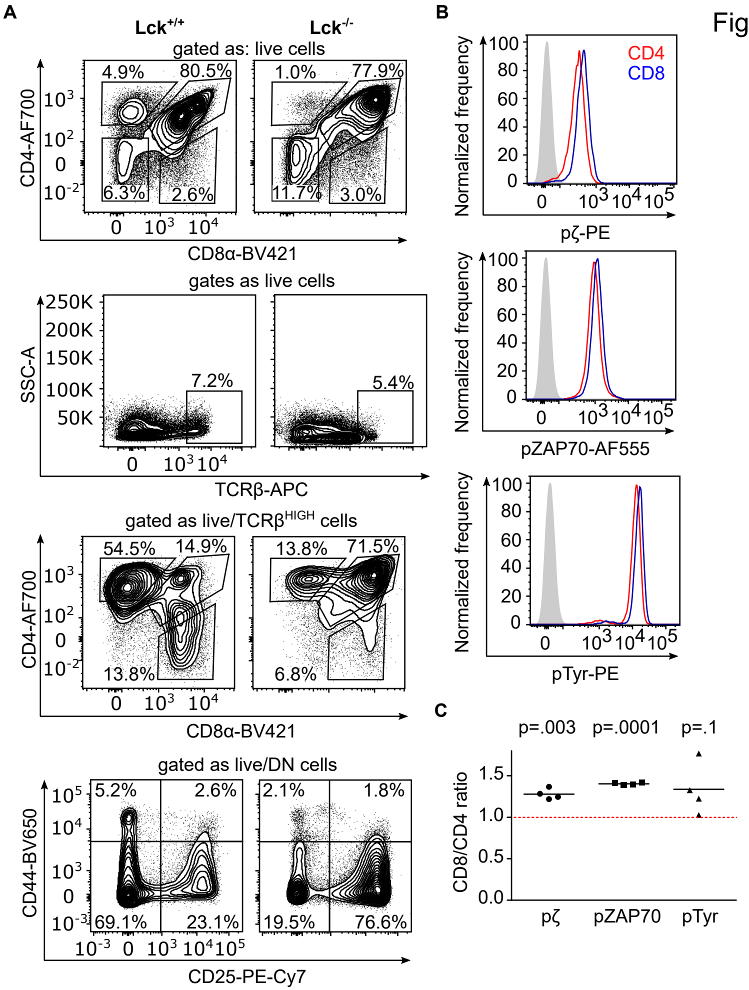

A
Lck+/+
Lck-/-
gated as: live cells
4.9%
80.5%
6.3%
2.6%
1.0%
77.9%
11.7%
3.0%
CD4-AF700
CD8α-BV421
gates as live cells
7.2%
5.4%
SSC-A
250K
200K
150K
100K
50K
TCRβ-APC
gated as live/TCRβHIGH cells
54.5%
14.9%
13.8%
13.8%
71.5%
6.8%
CD4-AF700
CD8α-BV421
gated as live/DN cells
5.2%
2.6%
69.1%
23.1%
2.1%
1.8%
19.5%
76.6%
CD44-BV650
CD25-PE-Cy7
B
CD4
CD8
Normalized frequency
pζ-PE
pZAP70-AF555
pTyr-PE
C
p=.003
p=.0001
p=.1
CD8/CD4 ratio
pζ
pZAP70
pTyr

Fig. S4. Related to Figure 4.

(A) Flow cytometry analysis of thymocytes from *Lck*$^{+/+}$ and *Lck*$^{-/-}$ mice using indicated antibodies and LIVE/DEAD near-IR staining. A representative experiment out of 3 in total. (B-C) Fixed and permeabilized LN T cells from *Foxp3*-GFP mice were stained with antibodies to CD4, CD8, TCRβ, pTCRζ, pZAP70, and overall tyrosine phosphorylation and analyzed by flow cytometry. Only GFP-negative cells are shown. A representative experiment out of 3 mice in 2 independent experiments in total.

Figure S5

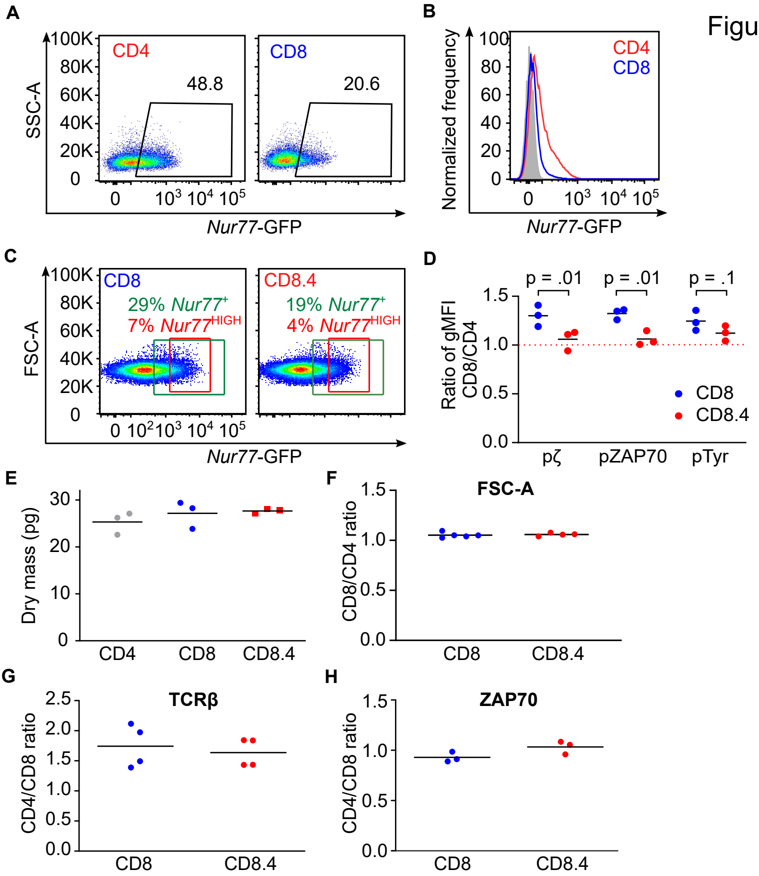

A
SSC-A
CD4
48.8
CD8
20.6
100K
80K
60K
40K
20K
0
0
10³ 10⁴ 10⁵
Nur77-GFP
B
Normalized frequency
CD4
CD8
100
80
60
40
20
0
0
10³ 10⁴ 10⁵
Nur77-GFP
C
FSC-A
CD8
29% Nur77⁺
7% Nur77^HIGH
CD8.4
19% Nur77⁺
4% Nur77^HIGH
0 10² 10³ 10⁴ 10⁵
Nur77-GFP
D
Ratio of gMFI CD8/CD4
p = .01
p = .01
p = .1
1.5
1.0
0.5
0.0
CD8
CD8.4
pζ
pZAP70
pTyr
E
Dry mass (pg)
30
20
10
0
CD4
CD8
CD8.4
F
FSC-A
CD8/CD4 ratio
1.5
1.0
0.5
0.0
CD8
CD8.4
G
TCRβ
CD4/CD8 ratio
2.5
2.0
1.5
1.0
0.5
0.0
CD8
CD8.4
H
ZAP70
CD4/CD8 ratio
1.5
1.0
0.5
0.0
CD8
CD8.4

Fig. S5. Related to Figure 5.

(A) Representative data from the $CD4^+$ and $CD8^+$ T cells gated for *Nur77*-GFP^+. Data out of 5 independent experiments are shown. (B) Representative data on *Nur77* expression of the $CD4^+$ and $CD8^+$ T cells. The data are the same as in Fig. S5A. (C) Representative data from CD8 WT and CD8.4 naïve T cells gated for *Nur77*-GFP^+ and *Nur77*-GFP^{HIGH}. (D) Independent repetition of experiment in Fig.5B performed in Prague. Statistical significance was calculated using paired T test. n = 3 mice in 3 independent experiments. (E) Peripheral LN T cells from C57Bl/6J and CD8.4 mice were analyzed on Tescan Q-Phase microscope. Dry mass of individual populations is displayed. Mean. n = 3 mice in 3 independent experiments. (F) Peripheral LN T cells from C57Bl/6J and CD8.4 mice were analyzed by flow cytometry and gated as $CD44^-$ $CD4^+$ or $CD44^-$ $CD8^+$ cells. The relative size (gMFI of FSC-A) of $CD8^+$ or $CD8.4^+$ cells to $CD4^+$ cells is displayed. Mean. n = 4-5 mice in 4-5 independent experiments. (G) Relative expression (gMFI) of *Zap70* in $CD8^+$ or $CD8.4^+$ T cells compared to $CD4^+$ T cells is shown. Mean. n = 3 mice in 3 independent experiments.

Table S1. Related to Fig. 1: Parameters for the mathematical model related to Fig.1C.

Parameter	diffusion (D)	coreceptor-MHC offrate (Ku)	lattice spacing (l)	coreceptor-MHC onrate (Kb)	pLck coupling (f)	number of TCRs/3 (T)	Area	k_{on}	k_p	phospho - rylation
Units	$\mu m^2 s^{-1}$	s^{-1}	µm	s^{-1}	%		μm^2	$\mu m^2 s^{-1}$	s^{-1}	
**CD4 DP thymocytes**	0.08	200	0.01	1000	0.051	697	26.18	0.1	5	5
**CD4 LN T cells**	0.08	200	0.01	1000	0.108	27861	26.18	0.1	5	5
**CD8 DP thymocytes**	0.08	20	0.01	1000	0.0054	697	26.18	0.1	5	5
**CD8 LN T cells**	0.08	20	0.01	1000	0.072	17203	26.18	0.1	5	5

Data S1 Related to Fig.1: Source code for ‘Lck come&stay/signal duration’ model (MatLab).

```
% Main script

% calculates Come%Stay model from given parametes using essentially 4
% conditions (can vary in coupling), the result are 4 matrices where X is
% DOSE and Y is dwell time)

%dwelltimes=zeros(100,100);
%for i=1:10 % makes an array of dwell times, always in columns from 0.05 to 20 in 0.025 steps, 1000 columns (all are the same)
    %dwelltimes(:,i)=i*0.1;
%end

%antigens=zeros(100,100); %makes an array of antigen numbers from 1 to 1000, 800 rows (all are the same)
%for j=1:100
    %antigens(j,:)=j;
%end

antigens=1:250;

sourcedata1=fopen('comenstaydata_5_6_P.txt','r'); %opens file with source data (dataDIAOTI.txt) and calls it sourcedata1
Dat=fscanf(sourcedata1,'%f', [10,4]) % reads data from the file
Cordata=Dat.';
Data1=Cordata(1,:) % condition 1
Data2=Cordata(2,:); % condition 2
Data3=Cordata(3,:); % condition 3
Data4=Cordata(4,:); % condition 4

dw1=0.86; % antigen dwell time (CD4s) 0.2 for threshold Ag, 0.86 FOR 3K
dw2=10.5; % antigen dwell time (CD8s) 0.9 for threshold Ag, 10.5 for OVA (approx. for thymus)

res1=dataprocess(Data1, dw1, antigens); % calls data process function for datasets 1-4
res2=dataprocess(Data2, dw1, antigens);
res3=dataprocess(Data3, dw2, antigens);
res4=dataprocess(Data4, dw2, antigens);

semilogy(antigens,res1, 'red:', 'LineWidth',2.5)
set(gca,'FontSize',17);

hold on % to draw all lines to the same graph
semilogy(antigens,res2, 'red', 'LineWidth',2.5)
semilogy(antigens,res3, 'blue:', 'LineWidth',2.5)
semilogy(antigens,res4, 'blue', 'LineWidth',2.5)
xlabel('Number of antigens','fontsize', 15, 'FontWeight','bold')
ylabel('# TCRs triggered','fontsize', 15, 'FontWeight','bold')
hold off
```

```
% Functions
function [lam] = markovchain ( D, Ku, l, Kb, f)
% Gives lambda parameter from an analytical Markov Chain solution
% lambda is the rate of Lck recruitment to the TCR via coreceptors
% Describes output of the coreceptor scanning mechanism
% D is a diffusion coefficient for membrane proteins, std 0.08 um^2
% x s-1
% cor_Koff is the correceptor Koff from the MHC molecule, std 20s-1 for
% CD8, 200s-1 for CD4
% l is a lattice spacing of the model, std 0.01 um
% cor_Kon is on-rate of the coreceptor-MHC interaction, std 1000s-1
lam = (f.*D.*Ku)./(D+l.^2.*Kb);
end

function [hotTCR] = dataprocess( dataValues, dwelltimeValue, antigenNumber )
% uses input data in a horizontal vector (reading from a file) and
% calculates the output of the comenstay, i.e. calculates lambda and then
% number of triggered and occupied TCRs

lambda=markovchain(dataValues(1,1), dataValues(1,2),dataValues(1,3), dataValues(1,4),dataValues(1,5))
hotTCR=comenstay(lambda, antigenNumber, dataValues(1,6), dataValues(1,7), dwelltimeValue, dataValues(1,8), dataValues(1,9), dataValues(1,10));

end

function [ R ] = comenstay( la, L, T, A, t, kon, kp, n )

%Gives abverage number of occupied and triggered TCRs at the equillibrium
%  from Stepanek et al. Cell 2014
% la is lambda (rate of Lck recruitment, calculated by markov chain model)
% L is number of antigen (pMHC) molecules) in the contact area, varied
% T is number of TCRs in the contact area
% A is the area of the T cell/APC inteface, std 26 um^2
% t is dwell-time, i.e. half-life (varied)
% kon is the on-rate of the antigen, std 0.1 um^2s-1
% kp is the phosphorylation rate, std 5s-1
% n is the required number of phosphorylation steps

%la=ones(100)*la1; %to have it in matrices (optional), change THE INPUT
%variables accordigly
%T=ones(100)*T1;
%A=ones(100)*A1;
%kon=ones(100)*kon1;
%kp=ones(100)*kp1;
%n=ones(100)*n1;
```

```
af=log(2)./(kon.*t); % Affinity, to simplify the final equation ln2/(kon x t)
koff=log(2)./t; % koff calculated

ProbTrig=la./(la+koff).*(kp./(kp+koff)).^n; %probability that a single TCR/MHC interaction leads to the TCR triggering
OccTCRs = (L/A+T/A+af-((L./A+T./A+af).^2-4*L.*T./A^2).^0.5)/2.*A; %average number of antigen occupied by MHC antigens in equillibrium, . removed when not necessary

R= ProbTrig.*OccTCRs; % number of triggered and occupied TCRs at the equilibrium, OUTPUT of the Come&Stay model
```
